# Catecholamines facilitate VEGF-dependent angiogenesis via β_2_-adrenoceptor-induced Epac1 and PKA activation

**DOI:** 10.18632/oncotarget.17267

**Published:** 2017-04-20

**Authors:** Jaspal Garg, Yu-Xi Feng, Sepp R. Jansen, Julian Friedrich, Frank Lezoualc'h, Martina Schmidt, Thomas Wieland

**Affiliations:** ^1^ Institute of Experimental and Clinical Pharmacology and Toxicology, Medical Faculty Mannheim, Heidelberg University, Mannheim, Germany; ^2^ 5th Medical Clinic, University Hospital Mannheim, Heidelberg University, Mannheim, Germany; ^3^ Institute of Cardiovascular and Metabolic Diseases, Inserm UMR-1048, Université Toulouse -Paul Sabatier, Toulouse, France; ^4^ Department of Molecular Pharmacology, Center of Pharmacy, University of Groningen, Groningen, The Netherlands

**Keywords:** angiogenesis, cAMP, VEGF, EPAC, PKA

## Abstract

Chronic stress has been associated with the progression of cancer and antagonists for β-adrenoceptors (βAR) are regarded as therapeutic option. As they are also used to treat hemangiomas as well as retinopathy of prematurity, a role of endothelial β_2_AR in angiogenesis can be envisioned. We therefore investigated the role of β_2_AR-induced cAMP formation by analyzing the role of the cAMP effector molecules exchange factor directly activated by cAMP 1 (Epac1) and protein kinase A (PKA) in endothelial cells (EC). Epac1-deficient mice showed a reduced amount of pre-retinal neovascularizations in the model of oxygen-induced retinopathy, which is predominantly driven by vascular endothelial growth factor (VEGF). siRNA-mediated knockdown of Epac1 in human umbilical vein EC (HUVEC) decreased angiogenic sprouting by lowering the expression of the endothelial VEGF-receptor-2 (VEGFR-2). Conversely, Epac1 activation by β_2_AR stimulation or the Epac-selective activator cAMP analog 8-p-CPT-2’-O-Me-cAMP (8-pCPT) increased VEGFR-2 levels and VEGF-dependent sprouting. Similar to Epac1 knockdown, depletion of the monomeric GTPase Rac1 decreased VEGFR-2 expression. As Epac1 stimulation induces Rac1 activation, Epac1 might regulate VEGFR-2 expression through Rac1. In addition, we found that PKA was also involved in the regulation of angiogenesis in EC since the adenylyl cyclase (AC) activator forskolin (Fsk), but not 8-pCPT, increased sprouting in Epac1-depleted HUVEC and this increase was sensitive to a selective synthetic peptide PKA inhibitor. In accordance, β_2_AR- and AC-activation, but not Epac1 stimulation increased VEGF secretion in HUVEC.

Our data indicate that high levels of catecholamines, which occur during chronic stress, prime the endothelium for angiogenesis through a β_2_AR-mediated increase in endothelial VEGFR-2 expression and VEGF secretion.

## INTRODUCTION

Angiogenesis is a complex process which includes migration and proliferation of endothelial cells (EC), establishment of a novel basement membrane and recruitment of peri-endothelial cells to the walls of the new vessels formed from pre-existing ones [1]. Pathological angiogenesis occurs in numerous diseases and is regulated by angiogenic factors and their receptors. Especially, the vascular endothelial growth factor (VEGF) – VEGF-receptor-2 (VEGFR-2) signaling cascade has been studied in detail. Its importance in pathological angiogenesis has been verified and this cascade is therapeutically targeted, for example in tumor angiogenesis [2].

Chronic stress, which results in high levels of circulating catecholamines, has been linked to the progression of cancer and to support tumor angiogenesis [3, 4]. G protein-coupled receptors (GPCRs) also contribute to the regulation of angiogenesis in EC [5–7]. Nevertheless, the β_2-_ adrenergic receptor (β_2_AR) which is the main isoform of endothelial β-adrenoceptors and induces cAMP production, has been linked to the lowering of endothelial permeability [8, 9]. Recent evidence from pre-clinical and clinical observations, indicate that β_2_AR- selective antagonists as well as non-subtype selective antagonists can ameliorate pathological angiogenesis in infantile hemangiomas and proliferative retinopathy [10–14]. Mechanistically, it was proposed that β_2_AR-antagonism counteracts the catecholamine - β_2_AR – cAMP – protein kinase A (PKA) driven VEGF production in several cell types including EC, especially under hypoxic conditions [10]. Besides the activation of PKA, cAMP activates another signaling pathway which is dependent on the exchange factor directly activated by cAMP (Epac). Epac proteins, Epac1 and Epac2, are guanine nucleotide exchange factors for the monomeric GTPase Rap. Epac1 is expressed in EC and its activation contributes to the above mentioned cAMP-dependent increase in endothelial tightness [15, 16]. Whether and how it contributes to the regulation of angiogenesis is still a matter of debate. Whereas one *in vitro* study reports its involvement in pro-angiogenic signaling [17], two other reports indicate involvement in anti-angiogenic pathways [18, 19].

We therefore investigated whether Epac1 is of importance for the β_2_AR- antagonist sensitive, oxygen-induced retinopathy (OIR) in mice [13], and analyzed the role of Epac1 as additional mediator of cAMP effects in angiogenic signaling in cultured EC. We provide evidence that Epac1 is regulating the expression and the amount of endothelial VEGFR-2 in a cAMP-dependent manner. In addition, we show that, together with the cAMP-PKA induced VEGF expression and secretion, the β_2_AR-cAMP-Epac1-dependent up-regulation of the VEGFR-2 is conditioning the endothelium for angiogenesis.

## RESULTS

### Epac1 deficient mice are less susceptible to oxygen-induced angiogenesis

**Figure 1:**
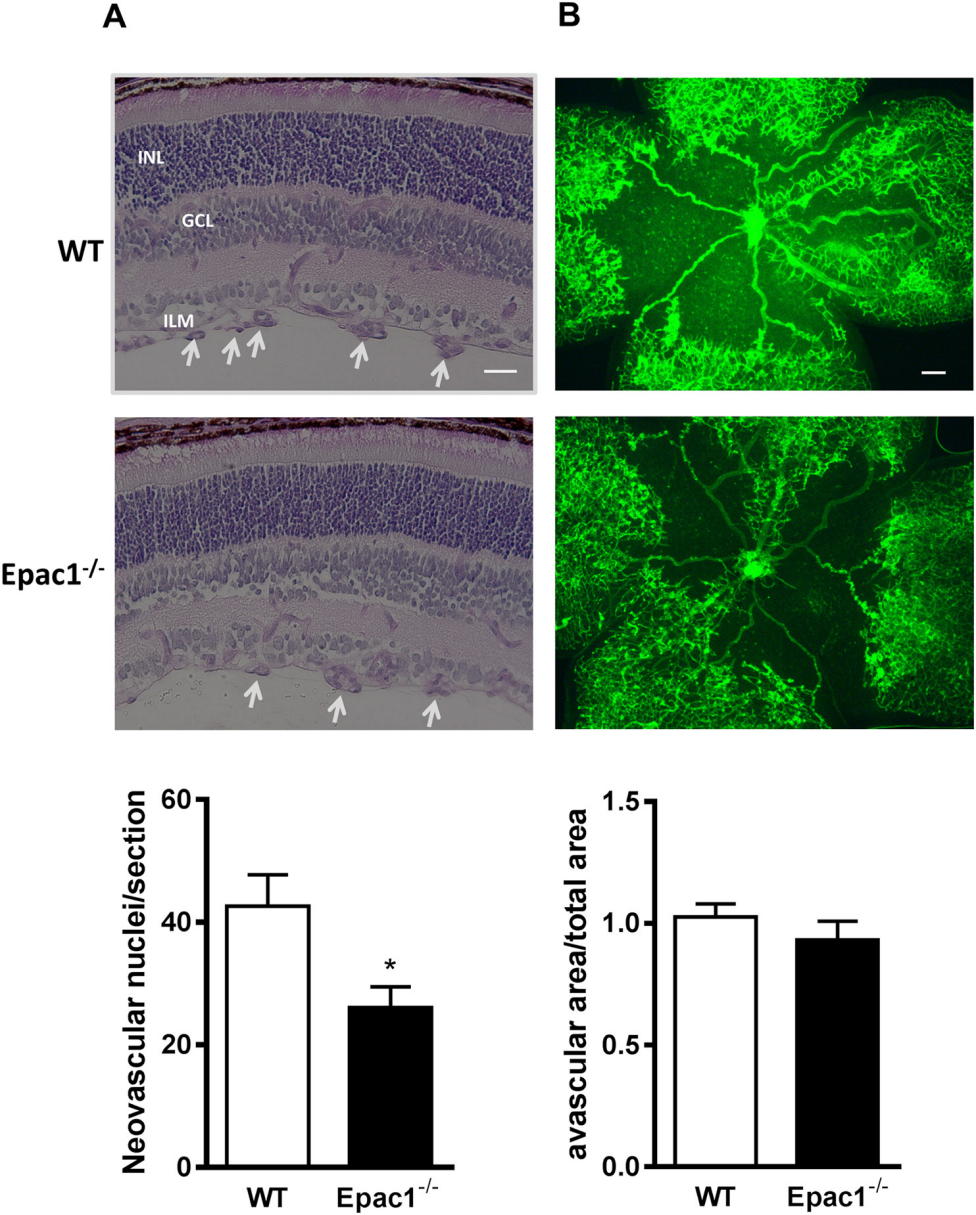
Retinae of Epac1^−/−^ mice are protected from oxygen-induced vascular damage New born pups at p7 along with their nursing mothers were put into 75% oxygen for 5 days and then returned to room air for another 5 days to induce relative hypoxia. At p17, mice were sacrificed, eyes enucleated and then fixed. **(A)** After embedding the eyes into paraffin, serial retinal sections were cut and stained with periodic acid and Schiff's base followed by hematoxylin. Representative pictures of one such retinal section from WT and its littermate Epac1^−/−^ mouse are shown. White arrowheads show the neovascular tufts. ILM: inner limiting membrane, GCL: ganglion cell layer, INL: inner nuclear layer. A quantification of neovascular endothelial nuclei originating from the ILM towards the vitreous per section per eye in WT and Epac1^−/−^ mice is shown below (n=13, Student's unpaired *t*-test, *p<0.01 *vs*. WT). **(B)** Retinae were dissected and subsequently stained with collagen IV antibody followed by staining with FITC-conjugated secondary antibody to visualize the retinal vasculature. Area of avascular zone relative to total retinal area in WT and Epac1^−/−^ mice from **(B)** to compare the levels of hypoxia present in the retina at p17 (n=7). Scale bar in **(A)**: 20 μm, in **(B)**: 200 μm.

In contrast to humans, which complete the maturation of the retinal vasculature at the time of birth, the vascular network in the mouse retina starts its development immediately after birth and reaches its mature state after three weeks of age. Therefore, the mouse retina is often used to study physiological angiogenesis in mammals. In addition, by subjecting the pups to the well-characterized OIR model [20, 21], also pathological angiogenesis, occurring for example in patients with diabetic retinopathy or human preterm infants suffering from retinopathy of prematurity [22], can be studied. Thus, to investigate whether Epac1 contributes to the regulation of vessel outgrowth in the retina, we analyzed physiological and pathological angiogenesis in Epac1-deficient (Epac1^−/−^) mice and control littermates [23]. Similar to control animals, Epac1^−/−^ mice displayed a normally developed retinal vasculature at postnatal day 5 (p5) and p17 (Supplementary Figure 1A-1B). Also, the morphology of the retinal arterioles was unchanged (Supplementary Figure 1B). Therefore, the data indicate no obvious role of Epac1 in the physiological angiogenesis and vessel development in the mouse retina. Since in the OIR model, the contribution of the β_2_AR to pathological angiogenesis has already been established [13, 24], we next subjected WT and Epac1^−/−^ mice to the OIR model. Consistent with previous studies [25], about 40 pre-retinal neovascularizations/section occurred in the retina of WT mice (Figure 1). In contrast, the number of pre-retinal neovascularizations was significantly reduced in Epac1^−/−^ mice (Figure 1A), despite the existence of similar levels of hypoxia in both groups (as judged by the formation of avascular zones, Figure 1B). These results therefore indicate an important contribution of Epac1 to the pathological, but not physiological angiogenesis in the mouse retina.

### Epac1 is involved in the regulation of angiogenic responses in HUVEC

**Figure 2:**
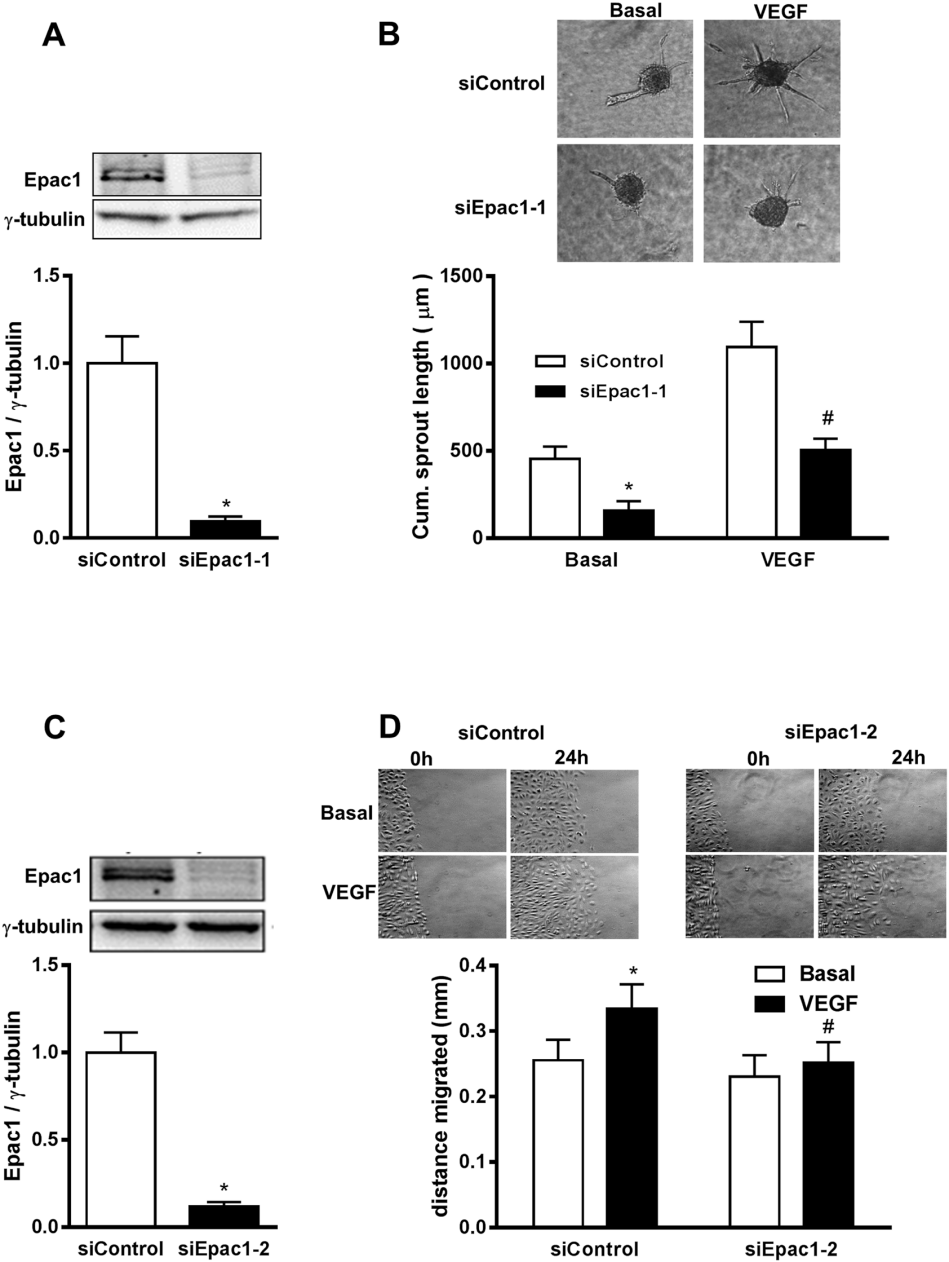
siRNA-mediated depletion of Epac1 in HUVEC impairs basal and VEGF-induced angiogenic responses **(A)** Sub-confluent HUVEC were either transfected with control siRNA or with siRNA against Epac1 (siEpac1-1). A representative Western blot is shown to detect the efficiency of knockdown 48 h post transfection (above). γ-tubulin was used as a loading control. Quantification of Epac1 bands relative to γ-tubulin using densitometric analysis is shown below (n=10, Student's paired t-test, *p<0.001 vs. siControl). **(B)** 24 h after transfection with the indicated siRNAs, sprouting assay was performed and spheroids were stimulated with or without 50 ng/ml of VEGF. Representative pictures are shown above. The mean of the cumulative sprout length measured from about 10 spheroids is shown below (n=5, Repeated measures of two-way ANOVA with Bonferroni's multiple comparison post-test, *p<0.05 vs. Basal siControl, ^#^p<0.05 vs. VEGF siControl). **(C)** is similar to A except that a different siRNA sequence against Epac1 (siEpac1-2) (n=39, Student's paired t-test, *p<0.001 vs. siControl) was used. **(D)** 48 h post transfection with the indicated siRNAs, a ‘scratch’ was created in HUVEC monolayer followed by stimulation with or without 50 ng/ml VEGF. The data show the migrated distance 24 h post stimulation (n=5, repeated measures of two-way ANOVA with Bonferroni's multiple comparison post-test, *p<0.05 vs. siControl Basal, #p<0.05 vs. VEGF siControl).

Epac1^−/−^ mice used in this study are deficient for Epac1 in all cell types. Therefore, phenotypic alterations observed in the retina of Epac1^−/−^ mice might originate from several retinal cell types. Nevertheless, the pathological angiogenesis in the OIR model is driven by VEGF and VEGFR-2 activation on EC [21]. Therefore, we studied the effect of an acute, siRNA (siEpac1-1) – mediated depletion of Epac1 in EC on spheroid sprouting angiogenesis in HUVEC, a well-established model to investigate the effects of pro- and antiangiogenic factors *in vitro*. As shown in Figure 2A-2B, an about 90% depletion of Epac1 significantly impaired basal and VEGF-induced sprouting indicating an involvement of Epac1 in the regulation of angiogenesis in EC. As a control for the specificity of the siRNA-mediated depletion, we used a second Epac1-specific siRNA (siEpac1-2) and studied sheet migration in the classical wound-healing assay as well as sprouting of HUVEC. The efficacy of siEpac1-2 to reduce Epac1 expression was similar to siEpac1-1 (Figure 2C) and Epac1 depletion by this siRNA significantly inhibited VEGF-induced sheet migration (Figure 2D) and basal as well as VEGF-induced sprouting (see Figure 7A).

**Figure 3:**
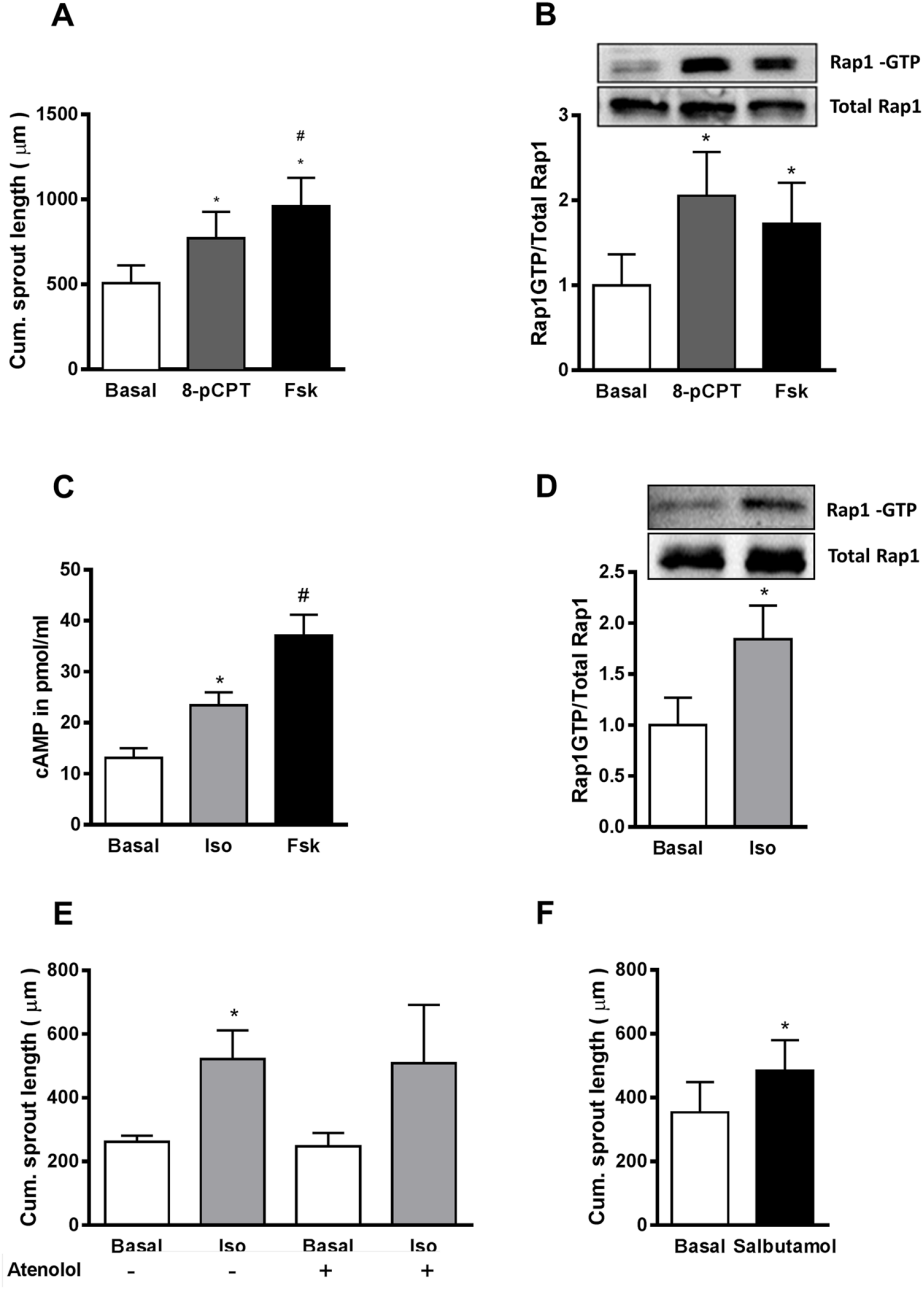
Activation of β_2_AR increases sprouting angiogenesis by cAMP-dependent activation of Epac1 **(A)** In the sprouting assay, HUVEC spheroids were stimulated with either 30 μM of the Epac activator (8-pCPT) or by 10 μM forskolin (Fsk) for 24 h. Subsequently, the cumulative sprout length was measured (n=5, one-way ANOVA with Bonferroni's multiple comparison post-test, *p<0.05 vs. Basal, ^#^p<0.05 vs. 8-pCPT). **(B)** Serum starved HUVEC were stimulated with either 30 μM 8-pCPT or 10 μM Fsk for 10 min, followed by an active Rap1-pull down assay. Subsequently, total and GTP-bound Rap1 were detected using Western blot analysis. A representative blot is shown in the above panel. For the quantification of the Rap1-GTP bands relative to total Rap1, densitometric analyses from the Western blots were performed (n=3, one-way ANOVA with Bonferroni's multiple comparison post-test, *p<0.05 vs. Basal). **(C)** After 20 min pre-treatment with 75 μM of IBMX, HUVEC were stimulated for 10 min with either 5 μM of isoproterenol (Iso) or 10 μM of Fsk. Cells were lysed and the cAMP content was measured by ELISA (n=5, one-way ANOVA with Bonferroni's multiple comparison test *p<0.05 vs. Basal, ^#^p<0.05 vs. Iso). **(D)** An active Rap1-pull down assay was performed as in B, after 10 min stimulation with 5 μM Iso. A representative blot and the quantification are shown (n=5, Student's paired t-test *p<0.05 vs. Basal). **(E)** Shows the effect of 5 μM Iso on the cumulative sprout length in the presence or absence of 10 μM atenolol (n=4-8, one-way ANOVA with Bonferroni's multiple comparison test, *p<0.05 vs. Basal without atenolol). **(F)** Shows the effect of 10 μM salbutamol on the cumulative sprout length (n=4, Student's paired t-test *p<0.05 vs. Basal).

To further characterize the role of Epac1 in sprouting angiogenesis, we treated HUVEC with an Epac-specific cell permeable cAMP analog, 8-p-CPT-2’-O-Me-cAMP (8-pCPT) and the adenylyl cyclase (AC) activator forskolin (Fsk). Both, Fsk- and 8-pCPT-treatment had no influence on the Epac1 protein content in HUVEC (data not shown). The efficacy of these treatments to activate Epac1 was controlled by quantifying the amount of Rap1-GTP by an effector pull-down assay (Figure 3B, Supplementary Figure 2). The stimulation of HUVEC spheroids with 8-pCPT or Fsk significantly increased the sprouting angiogenesis (Figure 3A). Similarly, the activation of Epac1 by 8-pCPT significantly increased sheet-migration in the wound-healing assay (Supplementary Figure 3). Taken together, all these results point towards an important role of Epac1 in the regulation of pro-angiogenic responses in EC.

### Stimulation of the β_2_-adrenoceptor increases sprouting angiogenesis in HUVEC

HUVEC, like other EC, express various G_s_ protein coupled receptors (G_s_PCRs) including βARs, predominantly of the β_2_ subtype [8], which upon agonist binding induce cAMP production and thus Epac1 activation [26]. We therefore stimulated HUVEC with the βAR agonist isoproterenol (Iso). As shown in Figure 3C–3D, Iso treatment indeed significantly increased cAMP levels and induced Epac1 activation (as determined by active Rap1 pull-down) in HUVEC. Similar to direct Epac1 activation, the treatment of HUVEC spheroids with Iso significantly increased sprouting angiogenesis (Figure 3E). As this increase was not sensitive to the β_1_AR antagonist atenolol, but was mimicked by the β_2_AR-specific agonist salbutamol (Figure 3F), we conclude that the β_2_AR is specifically involved in the regulation of angiogenic sprouting.

### Activation of PKA contributes to the cAMP-induced increase in sprouting angiogenesis by increasing VEGF expression and secretion

**Figure 4:**
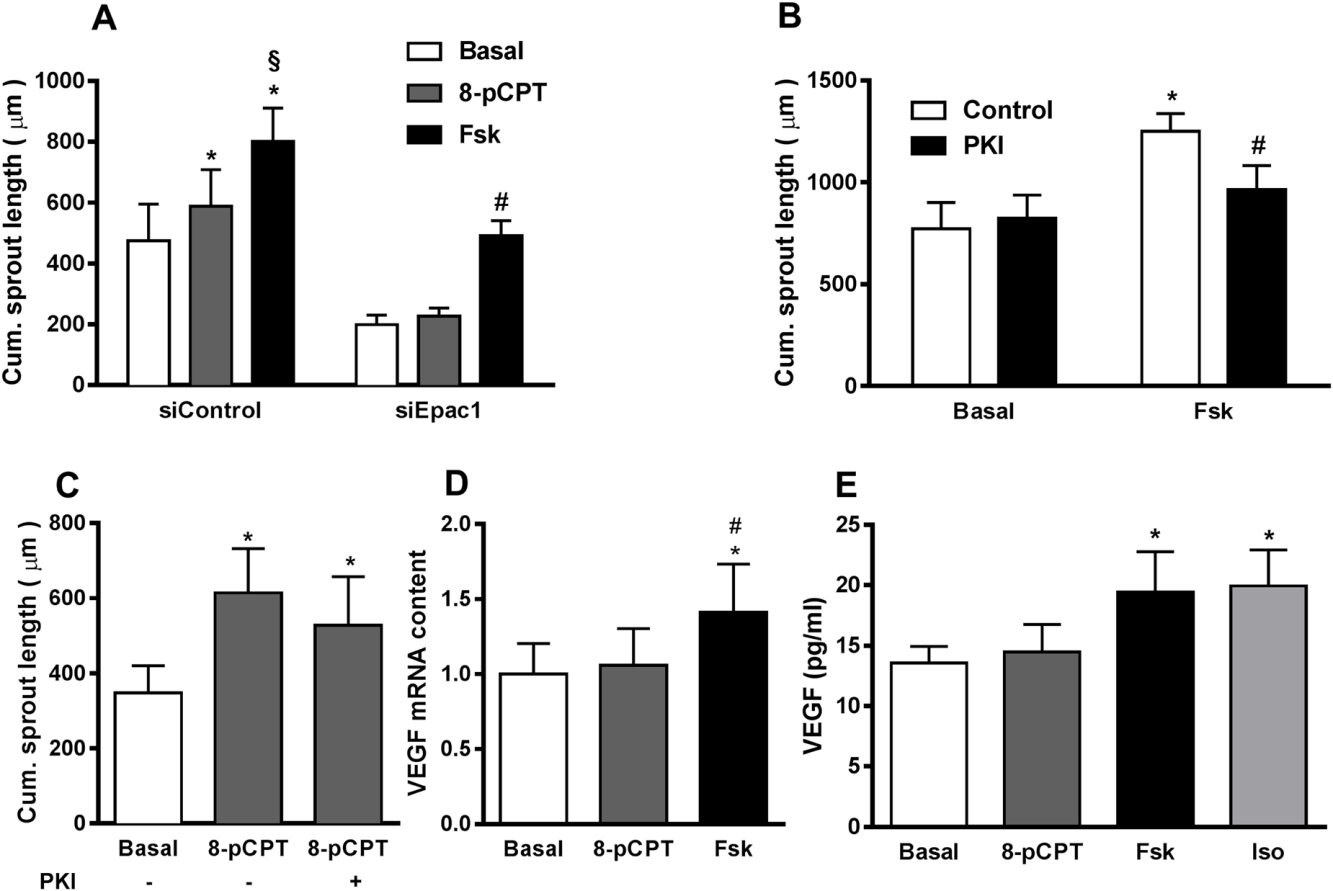
PKA contributes to cAMP-induced increase in sprouting angiogenesis **(A)** 24 h after transfecting HUVEC with either control siRNA or siRNA against Epac1, sprouting assays were performed in the presence or absence of either 30 μM of the Epac activator (8-pCPT) or 10 μM forskolin (Fsk). Subsequently, the cumulative sprout length was measured (n=10, repeated measures of two-way ANOVA with Bonferroni's multiple comparison post-test; *p<0.05 vs. siControl Basal, §p<0.05 vs. siControl 8-pCPT, ^#^p<0.05 vs. siEpac1 Basal). **(B)** In the sprouting assay, HUVEC spheroids were pre-treated with or without 1 μM of myristoylated-PKI (PKI) for 20 min followed by stimulation with or without 10 μM Fsk for 24 h. The cumulative sprout length was analyzed subsequently (n=4, two-way ANOVA with Bonferroni's multiple comparison test, *p<0.001 vs. Control Basal, ^#^p<0.05 vs. Fsk Control). **(C)** In the sprouting assay, after 20 min of pre-treatment with or without 1 μM of PKI, spheroids were stimulated with 30 μM 8-pCPT (n=5, one-way ANOVA with Bonferroni's multiple comparison test, *p<0.05 vs. Basal). **(D)** Serum starved HUVEC were stimulated with either 30 μM of 8-pCPT or 10 μM of Fsk. 8h post stimulation, the amount of VEGF mRNA was quantified relative to RPL10 mRNA by qPCR (n=6, one-way ANOVA with Bonferroni's multiple comparison test, *p<0.05 vs. Basal, ^#^p<0.05 vs. 8-pCPT **(E)** Serum starved HUVEC were stimulated with either 30 μM 8-pCPT, 10 μM Fsk or 5 μM Iso for 24 h. Amount of VEGF secreted in the media was then measured from the supernatants using ELISA (n=6, one-way ANOVA with Bonferroni's multiple comparison post-test, *p<0.05 vs. Basal).

As shown in Figure 3A, Fsk was more effective than 8-pCPT in increasing sprouting angiogenesis, although Epac1 activation by Fsk was weaker than by 8-pCPT (Figure 3B). As these data point towards a role of PKA in the cAMP-dependent regulation of sprouting in HUVEC, we compared the effect of Fsk and 8-pCPT on angiogenic sprouting with and without Epac1 depletion. As expected, 8-pCPT, which selectively activates Epac, was unable to increase sprouting in Epac1-depleted HUVEC. However, Fsk still increased sprout formation significantly in Epac1-depleted cells, suggesting the involvement of an additional non-Epac1 cAMP-effector, most likely PKA (Figure 4A). Accordingly, treatment of HUVEC with a selective myristoylated-PKA inhibitor peptide (PKI) significantly lowered Fsk-induced sprouting angiogenesis (Figure 4B). The Epac1-dependent sprouting in response to 8-pCPT was not attenuated by PKI, validating the specificity of this inhibitor as wells as of 8-pCPT in the spheroid based sprouting assay (Figure 4C). In line with these data, which show an involvement of PKA, a significant, Iso-induced increase in sprouting was still detectable in Epac1-depleted HUVEC although the Iso-stimulated Rap1 activation was completely suppressed (Supplementary Figure 4).

Secretion of VEGF is known to increase angiogenic sprouting in EC in an autocrine manner [27]. Moreover, a cAMP- and PKA-dependent induction of VEGF synthesis has been reported from a variety of cell types including HUVEC [28]. Therefore, we assessed the effect of Epac and PKA activation on the expression of VEGF by quantifying the VEGF mRNA in HUVEC. Interestingly, Fsk but not 8-pCPT increased the VEGF mRNA content indicating that PKA, but not Epac1, is involved in the regulation of VEGF transcription (Figure 4D). In line, Fsk and Iso, but not 8-pCPT significantly increased VEGF in the culture medium (Figure 4E). Taken together, these results demonstrate that besides Epac1, PKA activation contributes to cAMP-induced sprouting angiogenesis by enhanced production and secretion of VEGF.

### Epac1 and Rac1 activity similarly regulate VEGFR-2 expression

**Figure 5:**
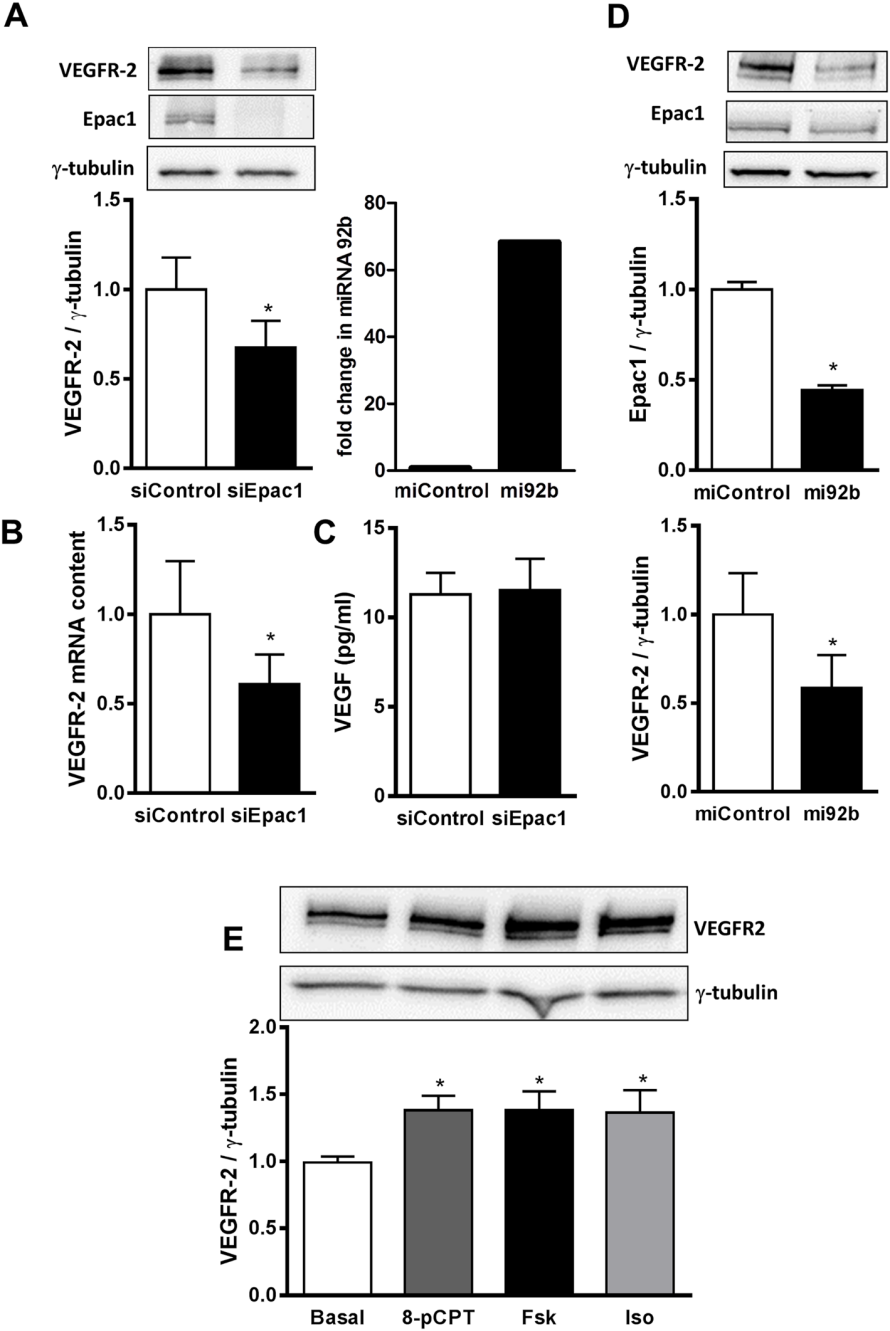
Epac1 regulates VEGFR-2 expression **(A)** HUVEC were transfected with control siRNA or siRNA against Epac1. 72 h post transfection, cells were lysed and VEGFR-2 and Epac1 protein levels were detected using Western blot analysis. γ-tubulin was used as a loading control (above). Densitometric analysis for the quantification of VEGFR-2 expression relative to γ-tubulin is shown below (n=6, Student's paired t-test, *p<0.01 vs. siControl). **(B)** 48 h post transfection with siRNAs, VEGFR-2 mRNA was quantified relative to RPL10 mRNA (n=4, Student's paired t-test, *p<0.05 vs. siControl). **(C)** After transfecting HUVEC either with control siRNA or siRNA against Epac1 for 48 h, the content of VEGF secreted in the media was determined using ELISA (n=3, Student's paired t-test). **(D)** HUVEC were transfected with control pre-miRNA or with pre-miRNA 92b. 48 h post transfection, cells were lysed and miRNA-92b levels were detected relative to U6 miRNA by qPCR to determine the efficiency of transfection (left). Right panel shows the Western blot analysis to detect Epac1 and VEGFR-2 protein levels. γ-tubulin was used as loading control (n=5, Student's paired t-test, *p<0.05 vs. miControl) **(E)** Serum starved HUVEC were stimulated for 24 h with either 30 μM 8-pCPT or 10 μM forskolin (Fsk) or 5 μM isoproterenol (Iso). Cells were lysed and Western blot analysis was performed to determine VEGFR-2 protein levels using anti-VEGFR-2 antibody. γ-tubulin was used as a house keeping protein. A representative blot is shown above. Densitometric analysis for the quantification of VEGFR-2 bands relative to γ-tubulin is shown below (n=7, one-way ANOVA with Bonferroni's multiple comparison post-test, *p<0.05 vs. Basal).

In line with the inability of 8-pCPT to increase VEGF expression, its mRNA content (Supplementary Figure 5) and secretion (Figure 5) were also unaffected by the depletion of Epac1 in HUVEC. We thus investigated whether Epac1 influences the amount of VEGFR-2, which mediates VEGF effects on endothelial cells [29]. Interestingly, the VEGFR-2 mRNA content was significantly reduced by about 50% in Epac1 depleted cells (Figure 5B). In line, the VEGFR-2 protein amount in HUVEC was also reduced by about 35% in Epac1 depleted cells (Figure 5A). Similar to HUVEC, the siRNA-mediated depletion of Epac1 in bovine aortic endothelial cells also decreased the VEGFR-2 levels and hence sprouting (Supplementary Figure 6). Furthermore, according to data base searches (TargetScan, RefGene), transcription of the Epac1 encoding gene *rapgef3* can be targeted by microRNA-92b (miR-92b). We therefore overexpressed this microRNA in HUVEC (Figure 5D) and analyzed the protein levels of Epac1 and VEGFR-2. The overexpression of miR-92b not only reduced Epac1 content by 55%, but also lowered the VEGFR-2 amount by 42%, indicating that a decrease in protein content of Epac1 by any approach (siRNA or miRNA), diminishes VEGFR-2 levels. We next studied whether the activation of Epac1 increases the VEGFR-2 protein levels. As shown in Figure 5E, treatment of HUVEC with 8-pCPT for 24 h significantly up-regulated the VEGFR-2 amount by about 40%. Interestingly, treatment with maximally stimulating concentrations of Fsk or Iso increased the VEGFR-2 protein level to a similar extent suggesting a role of Epac1, but not of PKA, in the up-regulation of VEGFR-2 expression.

**Figure 6:**
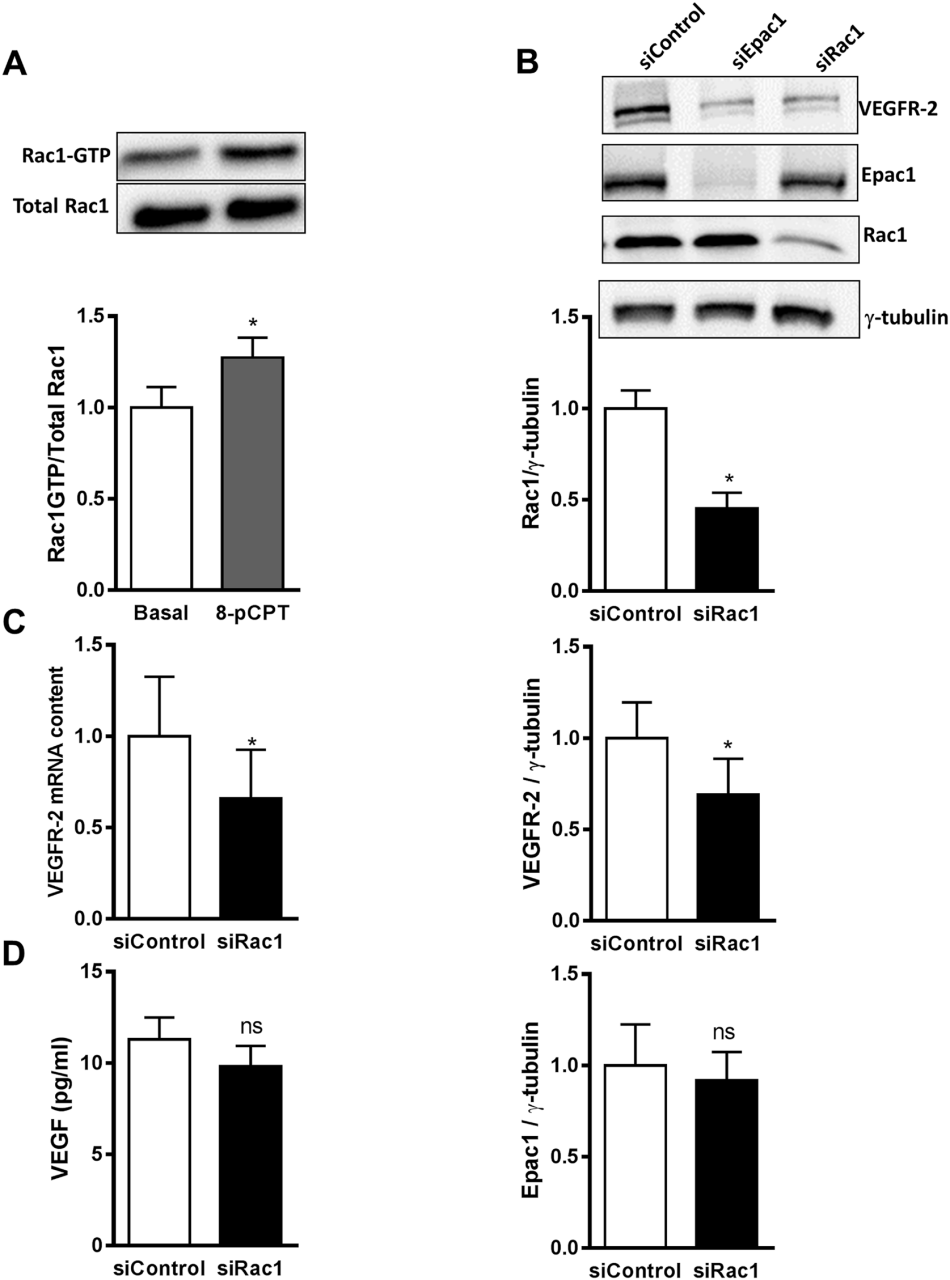
Rac1 as a mediator of Epac1-dependent VEGFR-2 expression **(A)** Serum starved HUVEC were stimulated with 30 μM 8-pCPT for 10 min. Subsequently, an active Rac1 pull-down assay was performed followed by Western blot analysis to detect GTP-bound and total Rac1. A representative blot is shown above. Densitometric analyses to quantify GTP-Rac1 band relative to total Rac1 are shown below (n=9, Student's paired t-test, *p<0.01 vs. Basal). **(B)** HUVEC were transfected with siControl or siRac1. 72 h post transfection, cell lysates were investigated for Rac1, VEGFR-2 and Epac1 proteins by Western blot analysis. γ-tubulin was used as a loading control. A representative blot is shown above. The protein content of Rac1, VEGFR-2 and Epac1 relative to γ-tubulin analysis was quantified by densitometry (n=4, Student's paired t-test, *p<0.05 vs. siControl, ns, p>0.05 vs. siControl). **(C)** HUVEC were transfected with either control siRNA or siRNA against Rac1. 48 h after transfection, cell lysates were analyzed for VEGFR-2 mRNA levels by qPCR. RPL10 mRNA was used as an endogenous control (n=4, Student's paired t-test, *p<0.05 vs. siControl) **(D)** 72 h after transfecting HUVEC with the indicated siRNAs, VEGF concentrations in the culture media were measured by ELISA (n=4, Student's paired t-test, ns, p>0.05 vs. siControl).

The monomeric GTPase Rac1 contributes to VEGF-induced EC migration by formation of lamellipodia and membrane ruffles [30]. In addition, it has been shown that Rac1 regulates VEGFR-2 expression in EC [31]. In accordance with data in the literature [15, 32], the selective activation of Epac1 increased the GTP-loading on Rac1 (Figure 6A) in an Epac-dependent manner (Supplementary Figure 7). We therefore tested, whether Rac1 knockdown decreases VEGFR-2 mRNA and protein levels in HUVEC to a similar extent as Epac1 depletion. As shown in Figure 6B, transfection of HUVEC with a validated Rac1-specific siRNA decreased Rac1 protein level by about 50% without any effect on Epac1 expression. Nevertheless, the decrease in Rac1 expression was accompanied by a significant loss of VEGFR-2 protein and mRNA by about 30% and 36%, respectively (Figure 6B–6C). Interestingly, the depletion of Rac1 affected neither VEGF transcription (data not shown) nor its secretion (Figure 6D). Taken together, these data indicate that the regulation of VEGFR-2 expression by Epac1 correlates with Rac1 activity in EC.

**Figure 7:**
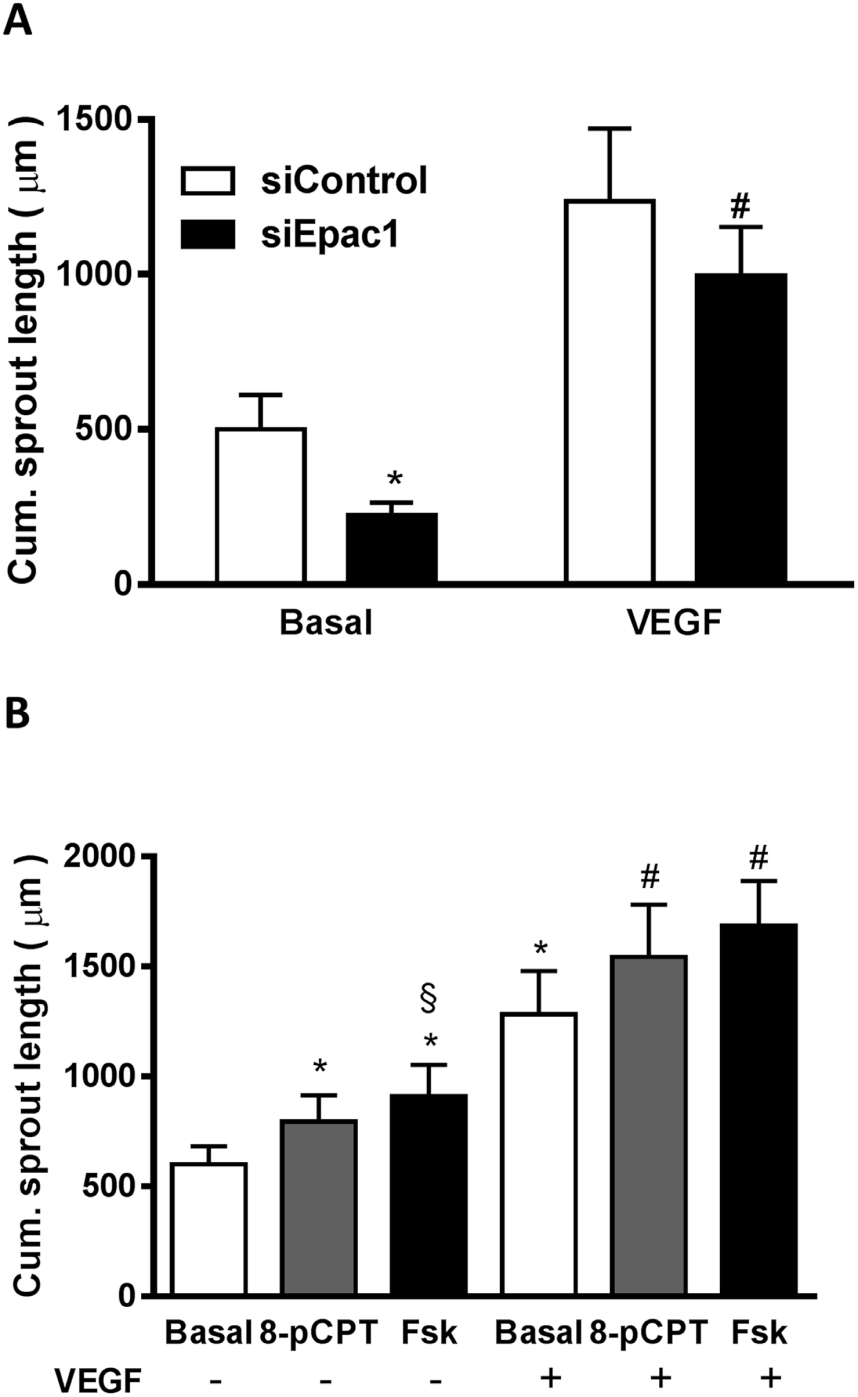
The Epac1-induced upregulation of VEGFR-2 is functionally relevant **(A)** 24 h after transfection with either siControl or siEpac1-2, sprouting assays were performed and the resultant spheroids were stimulated with or without 50 ng/ml VEGF. The data show the cumulative sprout length (n=8, repeated measures of two-way ANOVA with Bonferroni's multiple comparison post-test, *p<0.05 vs. Basal siControl, ^#^p<0.05 vs. VEGF siControl). **(B)** Spheroids were stimulated for 24 h with either 30 μM 8-pCPT or 10 μM forskolin (Fsk) in the absence or presence of 50 ng/ml VEGF. The cumulative sprout length was measured subsequently (n=8, one-way ANOVA with Bonferroni's multiple comparison test, *p<0.05 vs. Basal without VEGF, ^#^p<0.05 vs. Basal with VEGF, ^§^p<0.05 vs. 8-pCPT without VEGF).

### Regulation of the VEGFR-2 expression by Epac1 influences sprouting angiogenesis even in the presence of maximally stimulating concentrations of VEGF

To address the question whether the alterations in VEGFR-2 protein levels are functionally relevant even at saturating concentrations of VEGF, we analyzed basal and VEGF-stimulated (50 ng/ml) sprouting with and without Epac1 depletion (Figure 7A) or stimulation with 8-pCPT and Fsk (Figure 7B). Importantly, Epac1 depletion significantly decreased VEGF-induced sprouting angiogenesis. In accordance, the stimulation of Epac1 activity further enhanced the maximal VEGF-induced sprouting angiogenesis significantly. In contrast to the basal condition, there was however no significant difference in the effects of 8-pCPT and Fsk on the maximally VEGF-induced response which shows that the PKA-induced VEGF secretion is functionally irrelevant under this specific condition. Taken together, the data therefore indicate that the observed alterations in VEGFR-2 expression are of functional relevance even if high VEGF concentrations, originating for example from hypoxic conditions in a tumor, are present.

## DISCUSSION

### Dual role of Epac1 in the endothelium

Epac1 is the major Epac isoform expressed in the endothelium and its acute activation regulates endothelial permeability by influencing adherence and tight junction formation via the activation of the monomeric GTPases Rap1 and Rac1 [15, 16]. Indeed, we also observed a significant lowering of the permeability of an HUVEC monolayer upon the selective Epac1 activation (Supplementary Figure 8). A long term activation of Epac1 in the endothelium might however have a different outcome. It is therefore not surprising that divergent results have been reported, indicating either a pro-angiogenic [17, 33] or anti-angiogenic [18, 19] properties of endothelial Epac1, which are likely context dependent. To get an unbiased assessment whether Epac1 is generally involved in angiogenic responses, we studied physiological as well as pathological angiogenesis in the mouse retina. The OIR model was chosen because the involvement of the β_2_AR – cAMP system in the formation retinal neovascularizations has already been established [11, 13, 14, 24]. In line with the finding that the inhibition of the β_2_AR – cAMP cascade reduced the pathological angiogenesis in the retina by about 50% [13], Epac1 deficient mice showed a similar significant decrease in the occurrence of pre-retinal neovascularizations, indicating that Epac1 was part of the β_2_AR – cAMP cascade. In the *in vitro* model of cultured HUVEC, we obtained several lines of evidence that the Epac1-dependent regulation of the VEGFR-2 expression in EC was an important part in this response. Firstly, direct or indirect Epac1 activation increased VEGFR-2 levels to a similar extent and increased angiogenic sprouting even at saturating concentrations of VEGF. Secondly, depletion of Epac1 expression by different means (siRNA or miR92b-induced knockdown) severely diminished VEGFR-2 expression and hampered basal and VEGF-induced sprouting angiogenesis as well as sheet migration. Thirdly, the selective activation of Epac1 with 8-pCPT resulted in increased activation of the monomeric GTPase Rac1, the depletion of which decreased VEGFR-2 expression by a transcriptional mechanism [31] to a similar extent as Epac1 depletion. Therefore, our *in vivo* and *in vitro* data largely substantiate the disputed notion that Epac1, besides its well-known involvement in the regulation of endothelial barrier function, contributes to the cAMP-dependent regulation of angiogenesis in EC.

### The β_2_AR-cAMP-PKA/Epac1 cascade as a target in stress-conditioned angiogenesis

Chronic stress leads to constantly increased sympathetic tone with more circulating epinephrine and norepinephrine. High levels of catecholamines have been shown to increase VEGF production and secretion by activating the β_2_AR–cAMP–PKA pathway in a variety of cancer cells [3]. Therefore, chronic stress has been linked to the progression of cancer and especially non-subtype specific βAR antagonists, e. g. propranolol, are regarded as therapeutic option [4]. Likewise propranolol was used to cure infantile hemangiomas [34] and to prevent retinopathy of prematurity [35]. Our study provides a feasible explanation for the effectiveness of these treatments. Firstly, as reported before [27], cAMP-PKA -induced VEGF production and secretion occurs also in EC. Secondly, and more importantly, the cAMP-Epac1-dependent increase in VEGFR-2 is synergistically amplifying the response to β_2_AR stimulation, and thus likely priming the endothelium for angiogenesis. In line with our data, an earlier report indicated a synergistic action of PKA- and Epac1-dependent exocytosis of Weibel-Palade bodies in EC upon β_2_AR stimulation [33]. Thus approved unspecific beta-blockers, like propranolol or timolol, or β_2_AR-specific experimental ligands like ICI 118,551 [13] target important mechanisms, PKA-driven VEGF production and Epac1-mediated VEGFR-2 increase, by which β_2_AR stimulation regulates angiogenesis.

### Study limitations

We studied the effect of β_2_AR activation only in *in vitro* assays, as we could hereby ascertain that we can dissect the effects of cAMP-PKA and cAMP-Epac1 activation in EC. Although this limits the significance of our findings on the first sight, the evidence for a contribution of endothelial β_2_AR-cAMP signaling to pathological angiogenesis obtained from animal models and from clinical studies is impressive (for review see [10]). Therefore, we did not perform animal experiments using unspecific beta-blockers or specific β_2_AR antagonists. As an endothelial specific knockout of Epac1 is not available, we used in this study mice generally depleted in Epac1 [23]. Therefore, it is possible that in OIR model not only Epac1 depletion in EC, but also in other retinal cells contributed to the observed reduction in pre-retinal neovascularizations. Nevertheless, the similar effectiveness of Epac1 depletion and the inhibition of the β_2_AR [13] in the in the OIR model suggests that Epac1 is an important mediator of β_2_AR stimulation in this response. In line with this interpretation, Epac1 depletion in cultured EC had a rather strong effect on VEGFR-2 content and thus on sprouting and migration of EC.

### Conclusion and future perspectives

We could show herein that increasing cAMP by chronic activation of β_2_AR in EC apparently primes the endothelium for angiogenesis by activation of canonical PKA signaling which induces VEGF production and secretion as wells as an Epac1-dependent upregulation of VEGFR-2. Interestingly, stimulating angiogenesis *in vivo* is not unique to β_2_AR stimulation, but can also be achieved by other means of increasing endothelial cAMP, such as the inhibition of the cAMP degrading phosphodiesterase III [36] as well as a so far not well-characterized cAMP production inducing GPCR, i.e. GPR126 [37]. Although additional factors, like hepatocyte growth factor and angiopoietin1, might be involved in the cAMP-induced effects besides VEGF and VEGFR-2, these results clearly define the cAMP-PKA/Epac axis as molecular target for anti-angiogenic therapy. Exploring the therapeutic potential of Epac inhibitors [38, 39] in comparison to other options lowering the endothelial cAMP production is beyond the scope of this study, but can be envisioned.

The pharmacologic dissection of preclinical models of ovarian and prostate cancer revealed that catecholamine effects are mediated predominantly by β_2_ARs [40, 41]. In contrast to the more prescribed β_1_AR-selective drugs, propranolol was highly active in these models of tumor progression. Moreover, in addition to acting on the endothelium the interference with similar signaling pathways in cancer cells, for example non-small cell lung carcinoma (NSCLC) cells [42, 43], might be a reason why NSCLC patients benefit from the incidental use of beta-blockers for treatment of mainly cardiovascular diseases [44]. A pharmaco-epidemiologic analysis of breast cancer progression revealed however greater protective effects of propranolol than β_1_AR-selective agents [45]. Therefore, from a clinical point of view, the use of unspecific beta-blockers, like propranolol, which is generally well tolerated [10], might be the best option, currently. Nevertheless, clinical testing of more specific β_2_AR antagonists [13] for translation into humans might be worthwhile.

## MATERIALS AND METHODS

### Reagents

Forskolin (Fsk), isoproterenol (Iso), atenolol, salbutamol, 70 kDa FITC-Dextran and 3-Isobutyl-1-methylxanthine (IBMX) were purchased from Sigma-Aldrich. 8-p-CPT-2’-O-Me-cAMP (8-pCPT) and ESI-09 were from Biolog Life Sciences (Bremen, Germany). Recombinant human VEGF 165 was from PeproTech and myristyolated-PKA inhibitor peptide (PKI) from Enzo Life Sciences. All other reagents used were of analytical grade.

### Experiments with mice

Epac1^+/−^ mice were generated in the laboratory of Frank Lezoualc'h, University of Toulouse, France. Animal care and experiments were performed in accordance with the Association for Research in Vision and Ophthalmology (ARVO) statement and were approved by the local government (Regierungspräsidium Karlsruhe, Germany). All efforts were made to reduce the animal number as well as the animal suffering. Genotyping of the animals was performed as described previously [23].

### Oxygen-induced retinopathy (OIR)

Male and female Epac1^+/−^ mice were mated and the resultant new-born pups at p7, along with their nursing mothers, were placed into a chamber with 75% oxygen (PRO-OX-110, compact oxygen controller, BioSpherix) for 5 days to mimic hyperoxia. At p12, the mice were returned to room air for another 5 days to induce relative hypoxic conditions. New-born mice along with their mothers kept at room air during the entire length of the experimental period (p0-p17) were used as normoxic controls. At p17, the mice were sacrificed and the body weight was recorded. Eyes were then enucleated and analyzed for pre-retinal neovascularizations and whole mount retinal vessels visualizations. Only homozygous Epac1^−/−^ and their wild type (WT) littermates were analyzed in the study.

### Analysis of neovascularizations and the vasculature in the retinae

Quantification of neovascularizations was performed in the p17 eyes from the OIR model as described previously [46]. In brief, the eyes were fixed in 4% formalin overnight and then embedded into paraffin. Thereafter, serial sections of 6 μm were cut through the cornea and parallel to the optic nerve. The sections were stained with periodic acid-Schiff's (PAS) followed by hematoxylin. 10 sections around the optic nerve, with an interval of 12 μm, were selected and the endothelial nuclei originating from the inner limiting membrane towards the vitreous side were counted under the normal microscope (Carl Zeiss, 40x magnification). The mean of these 10 counted sections represents the average neovascular nuclei per section per eye. No neovascular cell nuclei were observed in the normoxic control mice.

To study the vascular morphology in the retinae, eyes were fixed in 4% PFA at RT for 2 h. Thereafter, the retinas were dissected and washed with PBS. The retinas were permeabilized and blocked for 1 h in a solution containing 1% BSA and 0.5% Triton X-100. After few washes with PBS, the retinas were incubated with either collagen IV (BP8017, Acris, 1:200) or FITC-conjugated lectin (L9381, Sigma-Aldrich, 1:50) at 4 °C overnight to visualize the retinal vessels. In case of collagen IV, FITC- (F0205, DAKO, 1:20) or TRITC-conjugated (R0156, DAKO, 1:20) swine anti-rabbit antibodies were used as secondary antibodies. Images were acquired with microscope (Olympus IX81) and the measurements of vascularized area (p5) and avascular zone (OIR p17) were performed by using Olympus Cell ^M^ software.

### Culturing of human umbilical vein endothelial cells (HUVEC)

HUVEC were isolated from human umbilical cords as described previously [47]. Cells were seeded on gelatin coated dishes and cultured in endothelial cell growth medium (Promocell, C-22110) containing supplements (Promocell, C-39210) and 2% (v/v) fetal calf serum (FCS) (Promocell, C-37350). Cells were kept under standard cell culture conditions (37 °C, 100% humidity, 5% CO2) and used at passage 2 to 4 for experiments.

### Transfection with small interfering RNAs (siRNAs) and micro RNAs (miRNAs)

HUVECs were transfected with either control siRNA (1027281, Qiagen) or siRNA targeting Epac1 (siEpac1-1, 5'-AACTCGGTGAAGCGAGAATTA-3, SI0069 8530, Qiagen; siEpac1-2, 5’-AGGGCACTTCGT GGTACATTA-3’, SI00698528, Qiagen), Rac1 (5’-ATGCATTTCCTGGAGAATATA-3’, SI02655051, Qiagen) at 60-70% confluency. Cells were transfected in 6-well plates using 250 pmol of the siRNAs per well. Transfection was performed according to the manufacturer's protocol (Lipofectamine^®^ RNAiMAX Transfection Reagent, Invitrogen, 13778-150). Similarly, miRNAs were over-expressed by transfecting HUVEC with 100 pmol of either the control miRNAs (Ambion, AM17110) or or miRNA-92b-3p (miR-92b, Ambion, PM10102) using the same transfecting reagent and scale. 48 hours after transfection, the cells were lysed in RIPA buffer (50 mM Tris-HCl, pH 7.4, 1% TritonX-100, 150 mM NaCl, 1 mM EDTA, 0.1% SDS) containing 1 mM of DTT, 1 mM of Na_3_VO_4_ and 0.1 mg/ml of Pefabloc^®^. Subsequently, proteins of interest were analyzed by Western blot analysis.

### Rap1 and Rac1 GTPase activation assay

The cellular level of active Rap1 and Rac1 were determined using effector pulldown assays [7, 48]. Briefly, the serum starved HUVEC were stimulated with indicated reagents for indicated time. The cells were lysed in ice-cold GST-Fish buffer (10% glycerol, 50 mM Tris–HCl, pH 7.4, 100 mM NaCl, 1% NP-40, 2 mM MgCl_2_). The GTP-bound small GTPases were precipitated with either Rap-binding domain of Ral or Rac-binding domain of Pak1 coupled to glutathione sepharose. The amounts of activated and total GTPases were then determined by Western blot analysis.

### Western blot analysis

HUVEC were lysed in RIPA buffer (as above) and proteins were separated in denaturing acrylamide gels and subsequently transferred to nitrocellulose membranes (Roche). After blocking with Roti^®^ Block (Carl Roth) for 1 h, the membranes were probed with primary antibodies overnight at 4 °C followed by incubation with secondary antibodies. The immuno-reactive bands were visualized with enhanced chemiluminescence (Thermo Fischer Scientific) and then quantified by the densitometry analysis using AlphaEase FC software (Alpha Innotech). Following primary antibodies were used for western blots: anti-Epac1 (4155, Cell Signaling, 1:500), anti-Rap1 (sc-65, Santa Cruz, 1:200), anti-Rap1a/Rap1B (4938, Cell Signaling, 1:1000), anti-Rac1 (610650, BD Biosciences, 1:1000), anti-VEGFR-2 (2479, Cell Signaling, 1:1000), anti-γ-tubulin (T6557, Sigma-Aldrich, 1:5000). Following secondary antibodies were used for Western blot: Horseradish peroxidase conjugated anti-mouse (A9044, Sigma-Aldrich, 1:20,000), Horseradish peroxidase conjugated anti-rabbit (A9169, Sigma-Aldrich, 1:80,000).

### Real-time PCR

Either serum starved HUVEC were stimulated for 8 h with the indicated substances or HUVEC were transfected with indicated siRNAs for 48 h. The cells were lysed in RLT Buffer (Qiagen), the total RNA was isolated (RNeasy Mini Kit, Qiagen) followed by synthesis of cDNA (SuperScript^®^ VILO cDNA Synthesis Kit, Thermo Fischer Scientific). Real-time PCR was performed in triplicates by using master mix (KK4705, Kapa Biosystems) and the Taqman probes for either VEGF (Hs00900055, Invitrogen) or VEGFR-2 (Hs00911700, Invitrogen), while RPL10 (Hs00749196, Invitrogen) was used as an internal control. A program of 50 °C x 2 min, 95 °C x 10 min followed by 40 cycles of 95 °C x 15 sec and 60 °C x 1 min was performed on Applied Biosystem's Step-One Plus Real-time PCR thermocycler in a 96-well plate format. Data were analyzed using the ΔΔCt method to determine the relative mRNA content.

### VEGF measurements

Either serum starved HUVEC were stimulated for 24 h with the indicated substances or HUVEC were transfected with indicated siRNAs for 48 h. The culture media were then collected and immediately transferred to liquid nitrogen for rapid freezing and stored at - 80 °C. VEGF concentrations were then measured by using a VEGF ELISA Kit (DVE00, R&D Systems, Abingdon, UK) following the manufacturer's instructions.

### cAMP measurements

Serum starved HUVEC were pre-treated for 20 min with 75 μM of IBMX to inhibit phosphodiesterase activity and subsequently stimulated with the indicated substances for 10 min. cAMP content was assessed by EIA Kit (BT900-066 Biotrend, Cologne, Germany) according to the manufacturer's instructions.

### Sprouting assay

*In vitro* sprouting angiogenesis assays with HUVEC were performed as described previously [49]. Briefly, 400 cells were mixed with methyl cellulose and spotted dropwise on a culture dish, which was then incubated up-side down for 24 h to generate the cellular spheroids. Spheroids were then embedded into a 3D collagen-based gel matrix pre-prepared from rat tail tendons followed by stimulation with the indicated reagents for 24 h. The cumulative sprout length was measured by microscopy (Olympus IX81, 10x magnification) using Olympus Cell^M^ software. In an individual experiment, around 10 spheroids were analyzed for each condition. In experiments involving siRNAs, the cells were transfected with the indicated siRNAs, 1 day prior to the generation of the spheroids.

### Sheet migration assay

Sheet migration assays were performed as previously described [50]. After overnight starvation of a fully confluent HUVEC monolayer, a ‘scratch’ was created with the help of a ‘stationery eraser’. Cells were washed several times to remove cellular debris and then were stimulated with indicated substances. Cells were maintained at 37 °C in a microscope stage-top incubator to enable time-lapse microscopy. The pictures were acquired every 30 min for 24 h (Olympus IX81) at two different locations on the edge of the scratch. The distance migrated was calculated by measuring the distance of the new edge from the starting edge from at least 10 different points by using the Olympus Cell ^M^ software.

### Permeability assay

Endothelial permeability was assayed by monitoring the passage of fluorescently labeled dextran through a tight monolayer of HUVEC grown to confluence. Briefly, HUVEC were seeded on the cell culture inserts (Thincerts, 1 μm, Greiner bio-tone). Next day, they were serum starved overnight followed by stimulation with 30 μM of 8-pCPT for 20 min and subsequent addition of 2 μM of 70 kDa dextran conjugated with on the top chamber. After 1 h, the extent of leakage of dextran was measured in a multi-label plate reader (EnVision 2102, Perkin Elmer) by measuring the fluorescence intensity (excitation at 490 nm, emission at 520 nm) from the lower chamber and then normalized to the fluorescence intensity in the respective upper chamber.

### Statistical analyses

The statistical analyses were performed using Graph Pad Prism version 6. The results are reported as mean ± SEM and the number of experiments performed (n) is described in the figure legends. Data of two groups and more than two groups were analyzed using the Student's t-test and one-way ANOVA with Bonferroni post-test, respectively. Data involving two or more independent factors were analyzed by two-way ANOVA with Bonferroni post-test. P values of ≤0.05 were considered statistically significant (*p ≤ 0.05).

## SUPPLEMENTARY MATERIALS FIGURES



## References

[1] Carmeliet P (2003). Angiogenesis in health and disease. Nat Med.

[2] Sitohy B, Nagy JA, Dvorak HF (2012). Anti-VEGF/VEGFR Therapy for Cancer: Reassessing the Target. Cancer Research.

[3] Cole SW, Sood AK (2012). Molecular pathways: beta-adrenergic signaling in cancer. Clin Cancer Res.

[4] Moreno-Smith M, Lutgendorf SK, Sood AK (2010). Impact of stress on cancer metastasis. Future Oncol.

[5] Benndorf R, Boger RH, Ergun S, Steenpass A, Wieland T (2003). Angiotensin II type 2 receptor inhibits vascular endothelial growth factor-induced migration and in vitro tube formation of human endothelial cells. Circ Res.

[6] Carbajo-Lozoya J, Lutz S, Feng Y, Kroll J, Hammes HP, Wieland T (2012). Angiotensin II modulates VEGF-driven angiogenesis by opposing effects of type 1 and type 2 receptor stimulation in the microvascular endothelium. Cell Signal.

[7] Del Galdo S, Vettel C, Heringdorf DM, Wieland T (2013). The activation of RhoC in vascular endothelial cells is required for the S1P receptor type 2-induced inhibition of angiogenesis. Cell Signal.

[8] Howell RE, Albelda SM, Daise ML, Levine EM (1985). Characterization of beta-adrenergic receptors in cultured human and bovine endothelial cells. J Appl Physiol.

[9] Spindler V, Waschke J (2011). Beta-adrenergic stimulation contributes to maintenance of endothelial barrier functions under baseline conditions. Microcirculation.

[10] Casini G, Dal Monte M, Fornaciari I, Filippi L, Bagnoli P (2014). The beta-adrenergic system as a possible new target for pharmacologic treatment of neovascular retinal diseases. Prog Retin Eye Res.

[11] Dal Monte M, Casini G, la Marca G, Isacchi B, Filippi L, Bagnoli P (2013). Eye drop propranolol administration promotes the recovery of oxygen-induced retinopathy in mice. Exp Eye Res.

[12] Filippi L, Cavallaro G, Bagnoli P, Dal Monte M, Fiorini P, Donzelli G, Tinelli F, Araimo G, Cristofori G, la Marca G, Della Bona ML, La Torre A, Fortunato P (2013). Oral propranolol for retinopathy of prematurity: risks, safety concerns, and perspectives. J Pediatr.

[13] Martini D, Monte MD, Ristori C, Cupisti E, Mei S, Fiorini P, Filippi L, Bagnoli P (2011). Antiangiogenic effects of beta2 -adrenergic receptor blockade in a mouse model of oxygen-induced retinopathy. J Neurochem.

[14] Ristori C, Filippi L, Dal Monte M, Martini D, Cammalleri M, Fortunato P, la Marca G, Fiorini P, Bagnoli P (2011). Role of the adrenergic system in a mouse model of oxygen-induced retinopathy: antiangiogenic effects of beta-adrenoreceptor blockade. Invest Ophthalmol Vis Sci.

[15] Baumer Y, Drenckhahn D, Waschke J (2008). cAMP induced Rac 1-mediated cytoskeletal reorganization in microvascular endothelium. Histochem Cell Biol.

[16] Kooistra MR, Corada M, Dejana E, Bos JL (2005). Epac1 regulates integrity of endothelial cell junctions through VE-cadherin. FEBS Lett.

[17] Namkoong S, Kim CK, Cho YL, Kim JH, Lee H, Ha KS, Choe J, Kim PH, Won MH, Kwon YG, Shim EB, Kim YM (2009). Forskolin increases angiogenesis through the coordinated cross-talk of PKA-dependent VEGF expression and Epac-mediated PI3K/Akt/eNOS signaling. Cell Signal.

[18] Doebele RC, Schulze-Hoepfner FT, Hong J, Chlenski A, Zeitlin BD, Goel K, Gomes S, Liu Y, Abe MK, Nor JE, Lingen MW, Rosner MR (2009). A novel interplay between Epac/Rap1 and mitogen-activated protein kinase kinase 5/extracellular signal-regulated kinase 5 (MEK5/ERK5) regulates thrombospondin to control angiogenesis. Blood.

[19] O'Leary AP, Fox JM, Pullar CE (2015). Beta-Adrenoceptor Activation Reduces Both Dermal Microvascular Endothelial Cell Migration via a cAMP-Dependent Mechanism and Wound Angiogenesis. J Cell Physiol.

[20] Smith LE, Wesolowski E, McLellan A, Kostyk SK, D'Amato R, Sullivan R, D'Amore PA (1994). Oxygen-induced retinopathy in the mouse. Invest Ophthalmol Vis Sci.

[21] Scott A, Fruttiger M (2009). Oxygen-induced retinopathy: a model for vascular pathology in the retina. Eye.

[22] Stahl A, Connor KM, Sapieha P, Chen J, Dennison RJ, Krah NM, Seaward MR, Willett KL, Aderman CM, Guerin KI, Hua J, Lofqvist C, Hellstrom A (2010). The mouse retina as an angiogenesis model. Invest Ophthalmol Vis Sci.

[23] Laurent AC, Bisserier M, Lucas A, Tortosa F, Roumieux M, De Regibus A, Swiader A, Sainte-Marie Y, Heymes C, Vindis C, Lezoualc'h F (2015). Exchange protein directly activated by cAMP 1 promotes autophagy during cardiomyocyte hypertrophy. Cardiovasc Res.

[24] Dal Monte M, Martini D, Latina V, Pavan B, Filippi L, Bagnoli P (2012). Beta-adrenoreceptor agonism influences retinal responses to hypoxia in a model of retinopathy of prematurity. Invest Ophthalmol Vis Sci.

[25] Feng Y, Gross S, Wolf NM, Butenschon VM, Qiu Y, Devraj K, Liebner S, Kroll J, Skolnik EY, Hammes HP, Wieland T (2014). Nucleoside diphosphate kinase B regulates angiogenesis through modulation of vascular endothelial growth factor receptor type 2 and endothelial adherens junction proteins. Arterioscler Thromb Vasc Biol.

[26] Mayati A, Levoin N, Paris H, N'Diaye M, Courtois A, Uriac P, Lagadic-Gossmann D, Fardel O, Le Ferrec E (2012). Induction of Intracellular Calcium Concentration by Environmental Benzo(a)pyrene Involves a β2-Adrenergic Receptor/Adenylyl Cyclase/Epac-1/Inositol 1,4,5-Trisphosphate Pathway in Endothelial Cells. Journal of Biological Chemistry.

[27] Takata K, Morishige K, Takahashi T, Hashimoto K, Tsutsumi S, Yin L, Ohta T, Kawagoe J, Takahashi K, Kurachi H (2008). Fasudil-induced hypoxia-inducible factor-1α degradation disrupts a hypoxia-driven vascular endothelial growth factor autocrine mechanism in endothelial cells. American Association for Cancer Research.

[28] Seya Y, Fukuda T, Isobe K, Kawakami Y, Takekoshi K (2006). Effect of norepinephrine on RhoA, MAP kinase, proliferation and VEGF expression in human umbilical vein endothelial cells. European Journal of Pharmacology.

[29] Kroll J, Waltenberger J (1997). The vascular endothelial growth factor receptor KDR activates multiple signal transduction pathways in porcine aortic endothelial cells. J Biol Chem.

[30] Soga N, Connolly JO, Chellaiah M, Kawamura J, Hruska KA (2001). Rac regulates vascular endothelial growth factor stimulated motility. Cell Commun Adhes.

[31] Meissner M, Michailidou D, Stein M, Hrgovic I, Kaufmann R, Gille J (2009). Inhibition of Rac1 GTPase downregulates vascular endothelial growth factor receptor-2 expression by suppressing Sp1-dependent DNA binding in human endothelial cells. Exp Dermatol.

[32] Aslam M, Tanislav C, Troidl C, Schulz R, Hamm C, Gunduz D (2014). cAMP controls the restoration of endothelial barrier function after thrombin-induced hyperpermeability via Rac1 activation. Physiol Rep.

[33] van Hooren KW, van Agtmaal EL, Fernandez-Borja M, van Mourik JA, Voorberg J, Bierings R (2012). The Epac-Rap1 signaling pathway controls cAMP-mediated exocytosis of Weibel-Palade bodies in endothelial cells. J Biol Chem.

[34] Sharma VK, Fraulin FO, Dumestre DO, Walker L, Harrop AR (2013). Beta-blockers for the treatment of problematic hemangiomas. Can J Plast Surg.

[35] Bancalari A, Schade R, Munoz T, Lazcano C, Parada R, Pena R (2016). Oral propranolol in early stages of retinopathy of prematurity. J Perinat Med.

[36] Sanada F, Kanbara Y, Taniyama Y, Otsu R, Carracedo M, Ikeda-Iwabu Y, Muratsu J, Sugimoto K, Yamamoto K, Rakugi H, Morishita R (2016). Induction of Angiogenesis by a Type III Phosphodiesterase Inhibitor, Cilostazol, Through Activation of Peroxisome Proliferator-Activated Receptor-gamma and cAMP Pathways in Vascular Cells. Arterioscler Thromb Vasc Biol.

[37] Cui H, Wang Y, Huang H, Yu W, Bai M, Zhang L, Bryan BA, Wang Y, Luo J, Li D, Ma Y, Liu M (2014). GPR126 protein regulates developmental and pathological angiogenesis through modulation of VEGFR2 receptor signaling. J Biol Chem.

[38] Courilleau D, Bouyssou P, Fischmeister R, Lezoualc'h F, Blondeau JP (2013). The (R)-enantiomer of CE3F4 is a preferential inhibitor of human exchange protein directly activated by cyclic AMP isoform 1 (Epac1). Biochem Biophys Res Commun.

[39] Zhu Y, Chen H, Boulton S, Mei F, Ye N, Melacini G, Zhou J, Cheng X (2015). Biochemical and pharmacological characterizations of ESI-09 based EPAC inhibitors: defining the ESI-09 “therapeutic window”. Sci Rep.

[40] Palm D, Lang K, Niggemann B, Drell TLt, Masur K, Zaenker KS, Entschladen F (2006). The norepinephrine-driven metastasis development of PC-3 human prostate cancer cells in BALB/c nude mice is inhibited by beta-blockers. Int J Cancer.

[41] Thaker PH, Han LY, Kamat AA, Arevalo JM, Takahashi R, Lu C, Jennings NB, Armaiz-Pena G, Bankson JA, Ravoori M, Merritt WM, Lin YG, Mangala LS (2006). Chronic stress promotes tumor growth and angiogenesis in a mouse model of ovarian carcinoma. Nat Med.

[42] Casibang M, Purdom S, Jakowlew S, Neckers L, Zia F, Ben-Av P, Hla T, You L, Jablons DM, Moody TW (2001). Prostaglandin E2 and vasoactive intestinal peptide increase vascular endothelial cell growth factor mRNAs in lung cancer cells. Lung Cancer.

[43] Jansen SR, Poppinga WJ, de Jager W, Lezoualc'h F, Cheng X, Wieland T, Yarwood SJ, Gosens R, Schmidt M (2016). Epac1 links prostaglandin E2 to beta-catenin-dependent transcription during epithelial-to-mesenchymal transition. Oncotarget.

[44] Wang HM, Liao ZX, Komaki R, Welsh JW, O'Reilly MS, Chang JY, Zhuang Y, Levy LB, Lu C, Gomez DR (2013). Improved survival outcomes with the incidental use of beta-blockers among patients with non-small-cell lung cancer treated with definitive radiation therapy. Ann Oncol.

[45] Barron TI, Connolly RM, Sharp L, Bennett K, Visvanathan K (2011). Beta blockers and breast cancer mortality: a population- based study. J Clin Oncol.

[46] Chang SH, Feng D, Nagy JA, Sciuto TE, Dvorak AM, Dvorak HF (2009). Vascular permeability and pathological angiogenesis in caveolin-1-null mice. Am J Pathol.

[47] Baudin B, Bruneel A, Bosselut N, Vaubourdolle M (2007). A protocol for isolation and culture of human umbilical vein endothelial cells. Nat Protoc.

[48] Schmidt M, Dekker FJ, Maarsingh H (2013). Exchange protein directly activated by cAMP (epac): a multidomain cAMP mediator in the regulation of diverse biological functions. Pharmacol Rev.

[49] Korff T, Augustin HG (1998). Integration of endothelial cells in multicellular spheroids prevents apoptosis and induces differentiation. J Cell Biol.

[50] Liang CC, Park AY, Guan JL (2007). In vitro scratch assay: a convenient and inexpensive method for analysis of cell migration in vitro. Nat Protocols.

