# Supplementary material for: Catecholamines facilitate VEGF-dependent angiogenesis via β2-adrenoceptor-induced Epac1 and PKA activation
Source: Oncotarget. 2017 Apr 20;8(27):44732–48. doi: 10.18632/oncotarget.17267 (PMC5546514; doi:10.18632/oncotarget.17267)
Supplement: Supplementary file 1 [file oncotarget-08-44732-s001.pdf]

Catecholamines facilitate VEGF-dependent angiogenesis via β_2-adrenoceptor-induced Epac1 and PKA activation

SUPPLEMENTARY FIGURES

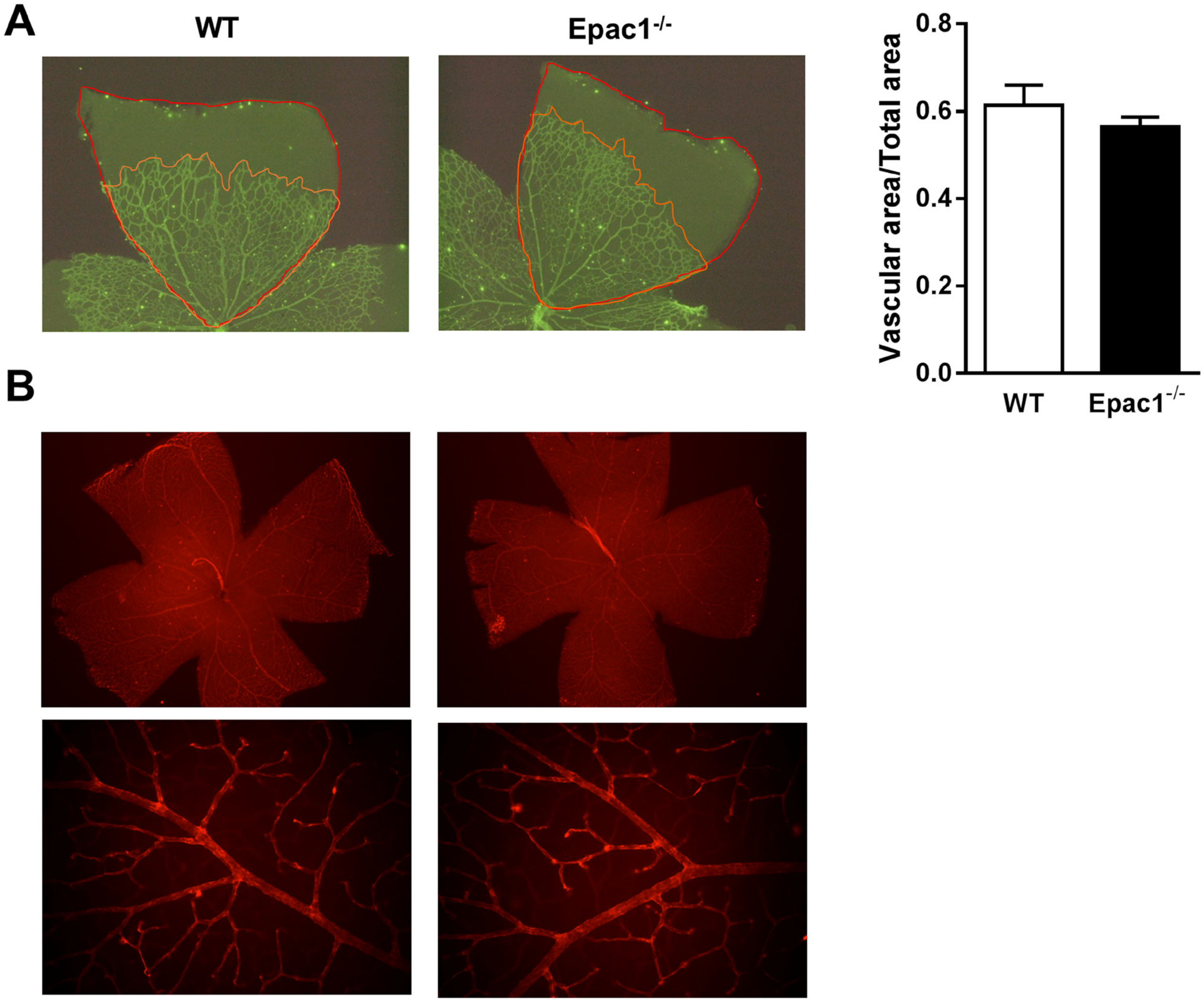

Supplementary Figure 1: WT and Epac1$^{-/-}$ mice display a similar physiological angiogenesis in the retina. Newborn pups were sacrificed at p5 and p17, eyes enucleated and fixed. Retinae were dissected and subsequently stained with collagen IV antibodies followed by FITC- or TRITC-conjugated secondary antibodies to visualize the retinal vasculature. **(A)** Representative pictures of the retinal vasculature from WT and Epac1$^{-/-}$ mice at p5. The red line encloses the total area whereas the orange line encloses the vascularized area. Ratio of vascularized area to the total area in the retina is shown on the right (n=7) **(B)** Representative pictures of the retinal vasculature (above) and the morphology of retinal arterioles (below) of WT and Epac1$^{-/-}$ mice at p17.

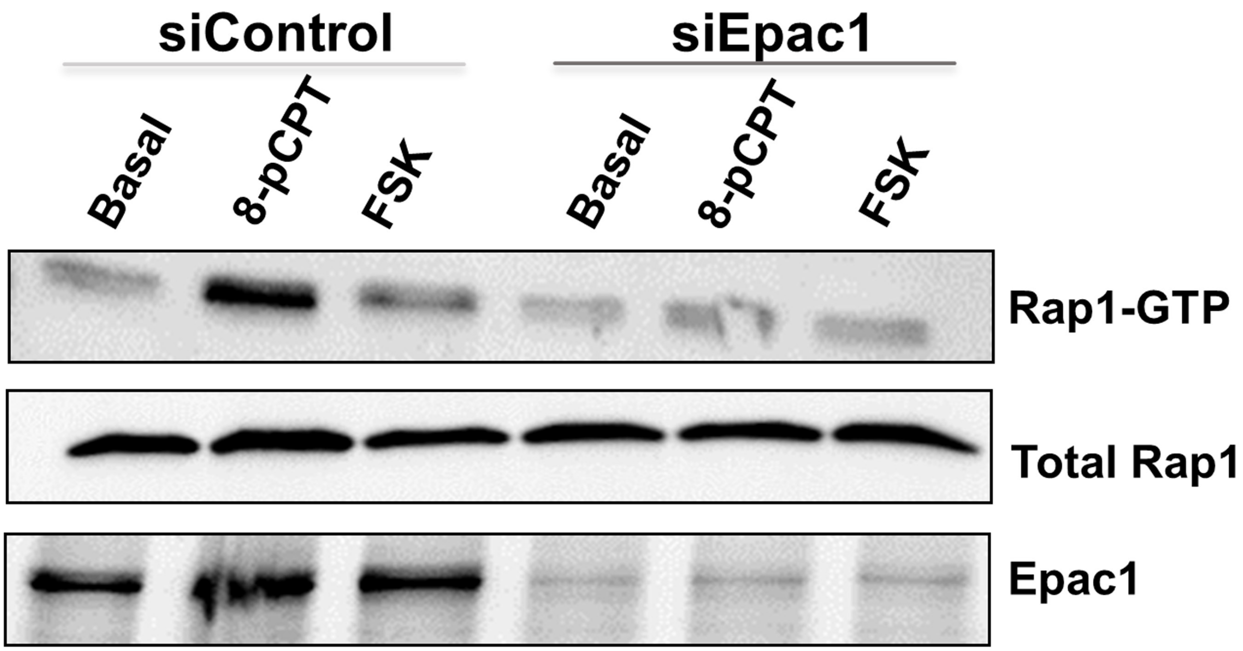

Supplementary Figure 2: Forskolin- and 8-pCPT-induced Rap1 activation is mediated by Epac1. HUVEC were transfected with either control siRNA or siRNA against Epac1. 48 h after transfection, cells were stimulated with either 30 µM 8-pCPT or 10 µM FSK for 10 min. Subsequently, an active Rap1 pull-down assay was performed followed by Western blot analysis to detect GTP-bound Rap1, total Rap1 and Epac1.

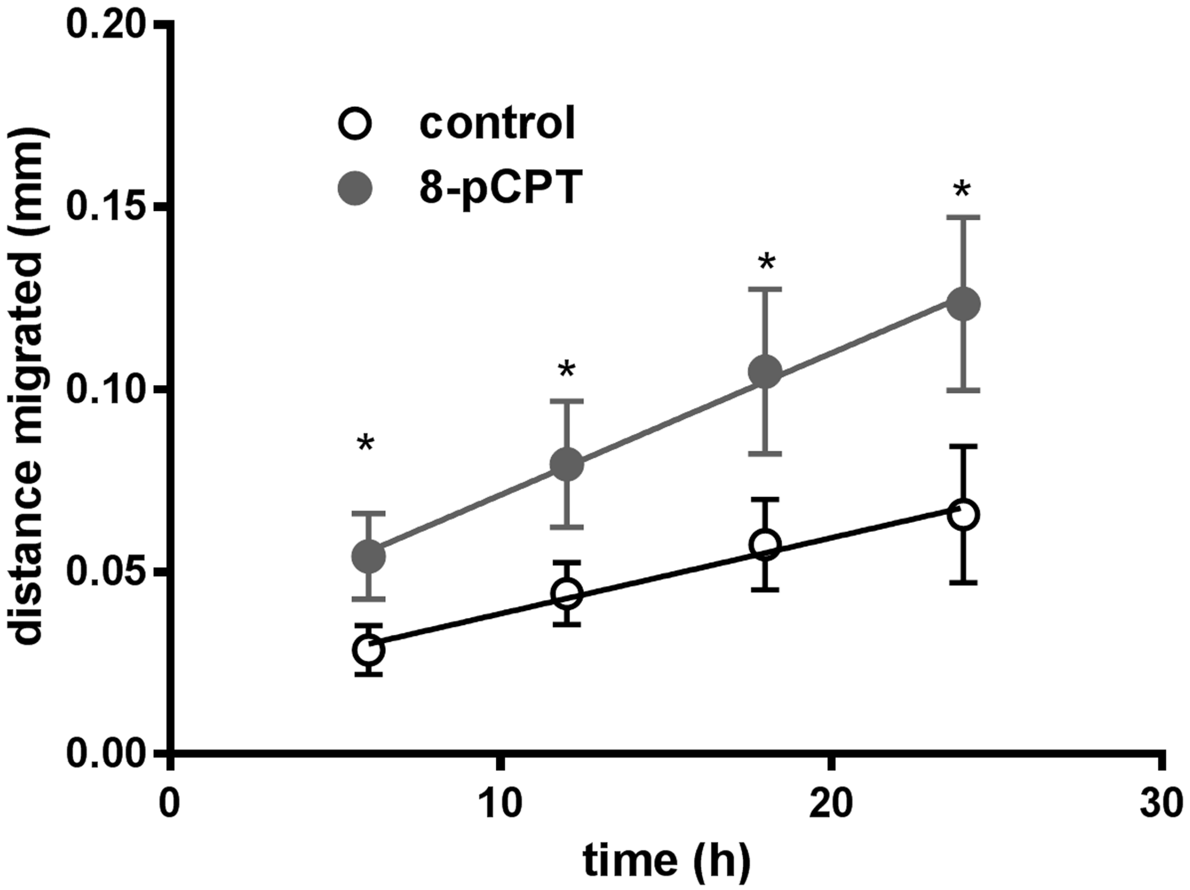

Supplementary Figure 3: Activation of Epac1 increases the velocity of sheet-migration. A ‘scratch’ was created in a fully confluent HUVEC monolayer followed by stimulation with and without 30 µM 8-pCPT. Pictures were taken every 30 min for 24 h under the time-lapse microscope. Data show the migrated distance with time (n=4, linear regression with Student’s paired *t*-test, *p<0.05 *vs.* control).

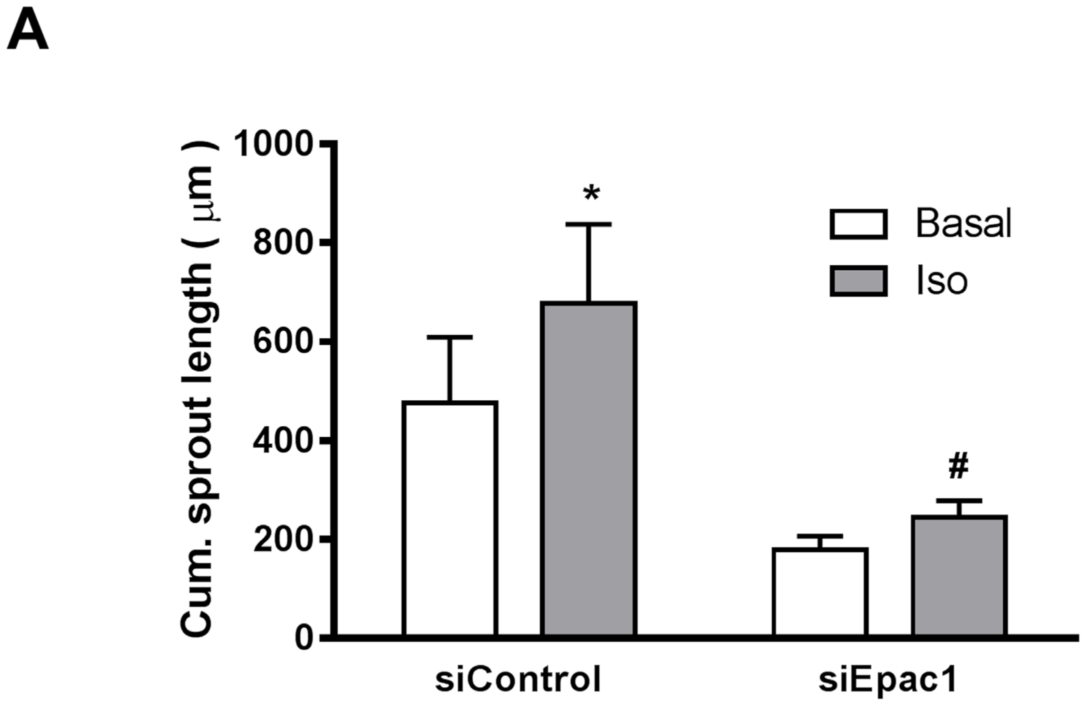

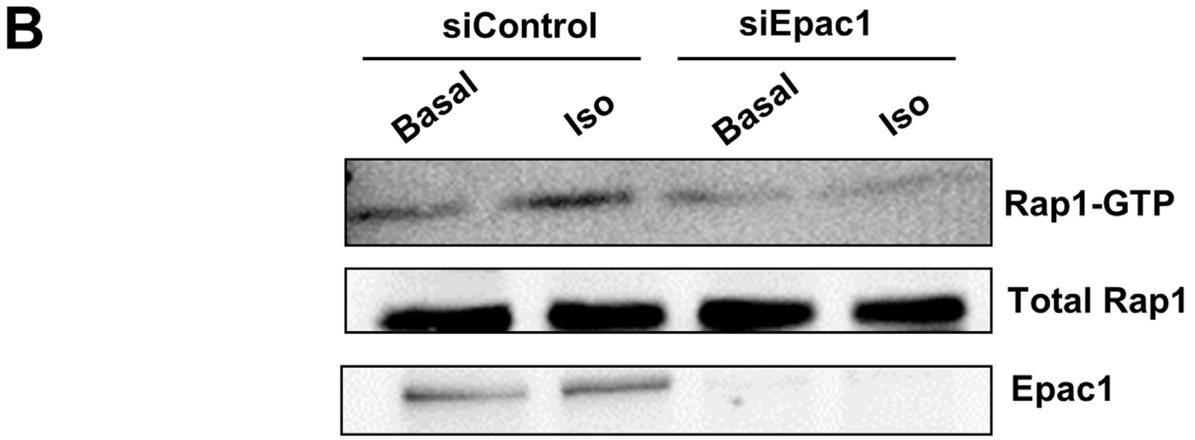

Supplementary Figure 4: Iso-induced increase in sprouting is still detectable after depletion of Epac1. **(A)** 24 h after transfecting HUVEC with the indicated siRNAs, the sprouting assay was performed. The spheroids were stimulated with or without 5 μM Iso and subsequently the cumulative sprout length was measured (n=6, one-way ANOVA with Bonferroni's multiple comparison test, $^{*}p<0.05$ vs siControl Basal, $^{\#}p<0.05$ vs siEpac1 Basal). **(B)** After transfecting HUVEC with indicated siRNAs for 48 h, Rap1 pull-down assay was performed with or without stimulation of 5 μM Iso for 10 min. Thereafter, Rap1 an Epac1 were detected using Western blot analysis. A representative blot is shown here.

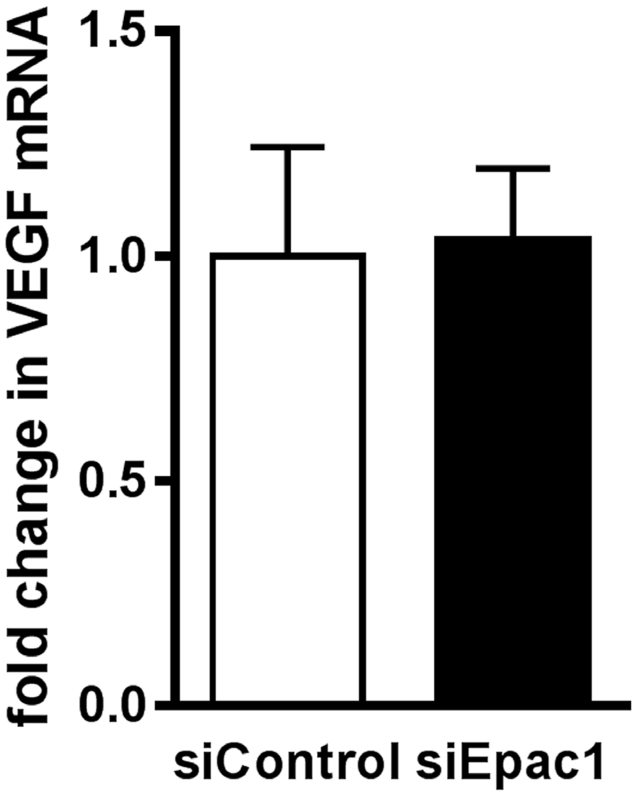

Supplementary Figure 5: Depletion of Epac1 does not alter VEGF mRNA content in HUVEC. 48 h post transfection with siRNAs, VEGF mRNA was quantified relative to RPL10 mRNA (n=5).

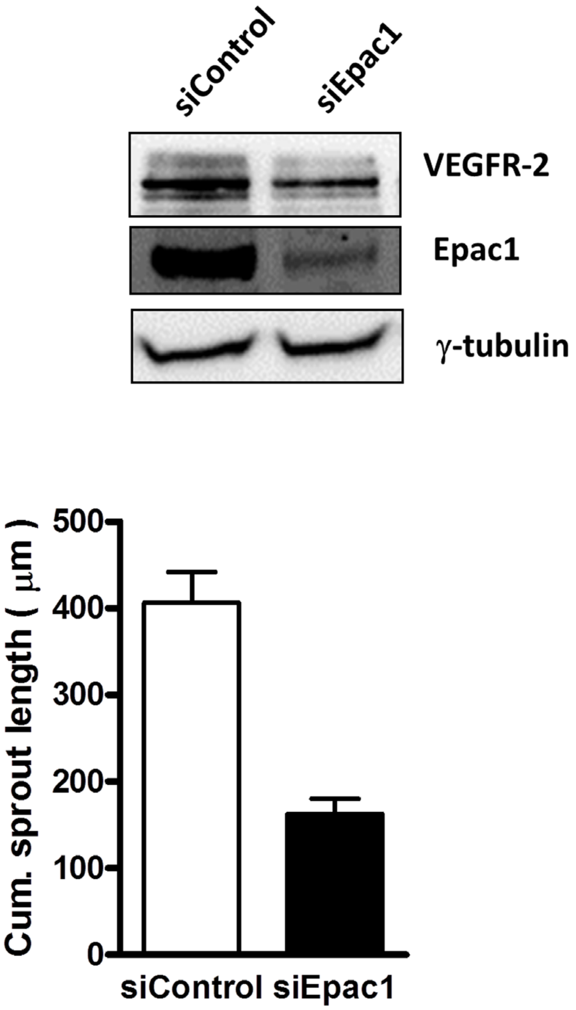

Supplementary Figure 6: Depletion of Epac1 in bovine aortic EC decreases VEGFR-2 content and sprouting. Bovine aortic EC were transfected with either siControl or siEpac1. 48h post transfection, Western blot analysis was performed to determine the VEGFR-2 levels. γ-tubulin was used as a loading control. 24 h after transfection with siRNAs, sprouting assay was performed and cumulative sprout length was measured.

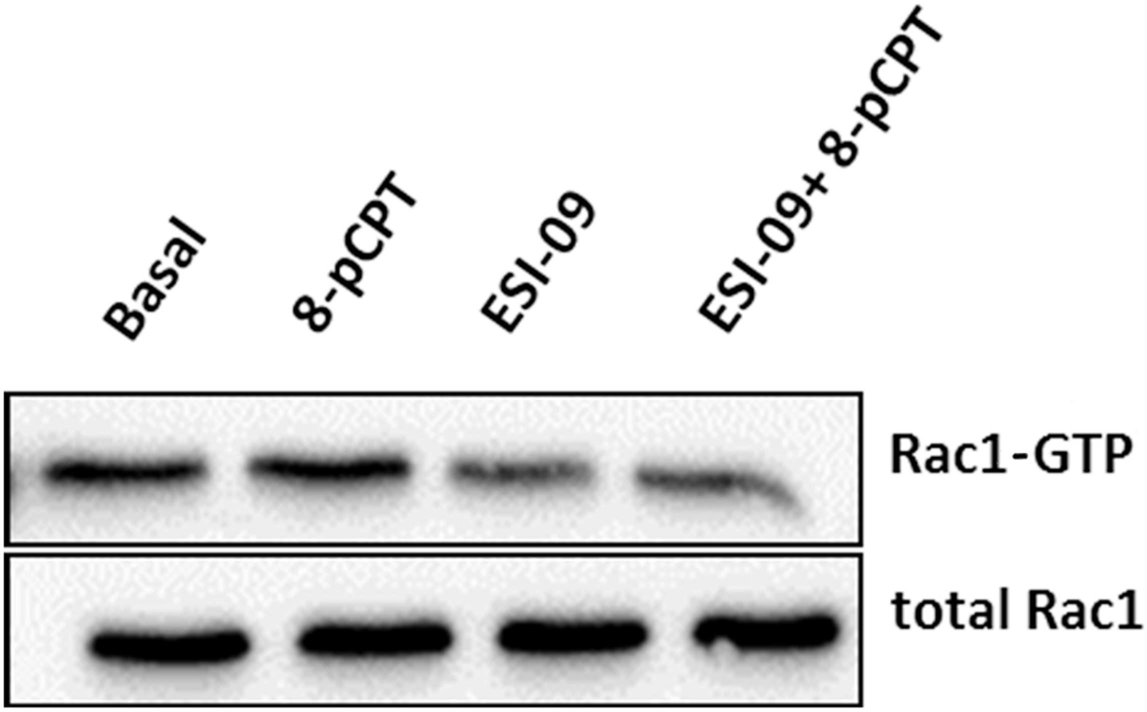

Supplementary Figure 7: Epac GEF activity is required for 8-pCPT-induced Rac1 activation. Serum starved HUVEC were stimulated with 30 µM 8-pCPT in the absence and presence of 5 µM of the Epac inhibitor ESI-09 for 10 min. Subsequently, an active Rac1 pull-down assay was performed followed by Western blot analysis to detect GTP-bound and total Rac1. A representative blot is shown

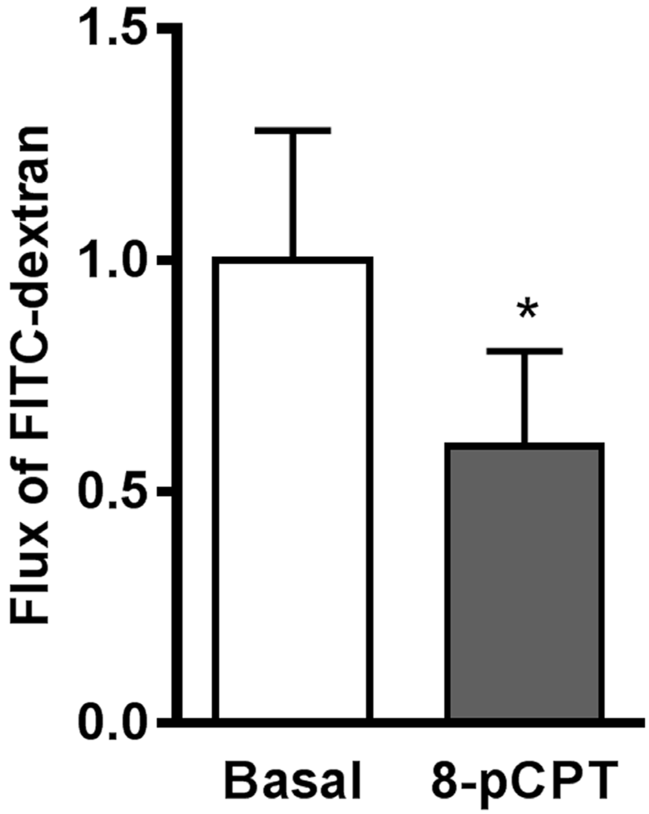

Supplementary Figure 8: Activation of Epac increases endothelial tightness. The flux of FITC-dextran was measured through a confluent monolayer of HUVEC in the permeability assay. Cells were stimulated with 30 µM 8-pCPT for 10 min followed by addition of 2 µM 70 kDa FITC-labeled dextran into the upper chamber. Data show the fluorescence intensity in the lower chamber normalized to the fluorescence intensity in the respective upper chamber after 1 h of addition of FITC-dextran (n=4, Student's paired *t*-test, *p<0.05 *vs*. Basal).
